# Transcriptional landscape and clinical utility of enhancer RNAs for eRNA-targeted therapy in cancer

**DOI:** 10.1038/s41467-019-12543-5

**Published:** 2019-10-08

**Authors:** Zhao Zhang, Joo-Hyung Lee, Hang Ruan, Youqiong Ye, Joanna Krakowiak, Qingsong Hu, Yu Xiang, Jing Gong, Bingying Zhou, Li Wang, Chunru Lin, Lixia Diao, Gordon B. Mills, Wenbo Li, Leng Han

**Affiliations:** 1 Department of Biochemistry and Molecular Biology, McGovern Medical School at The University of Texas Health Science Center at Houston, Houston, TX 77030 USA; 2 Department of Molecular and Cellular Oncology, The University of Texas MD Anderson Cancer Center, Houston, TX 77030 USA; 3 State Key Laboratory of Cardiovascular Disease, Fuwai Hospital, National Center for Cardiovascular Diseases, Chinese Academy of Medical Sciences and Peking Union Medical College, Beijing, 100037 PR China; 4 Department of Bioinformatics and Computational Biology, The University of Texas MD Anderson Cancer Center, Houston, TX 77030 USA; 5 Knight Cancer Institute, Oregon Health and Science University, Portland, OR 97239 USA; 6 Center for Precision Health, The University of Texas Health Science Center at Houston, Houston, TX 77030 USA

**Keywords:** Cancer genomics, Non-coding RNAs

## Abstract

Enhancer RNA (eRNA) is a type of noncoding RNA transcribed from the enhancer. Although critical roles of eRNA in gene transcription control have been increasingly realized, the systemic landscape and potential function of eRNAs in cancer remains largely unexplored. Here, we report the integration of multi-omics and pharmacogenomics data across large-scale patient samples and cancer cell lines. We observe a cancer-/lineage-specificity of eRNAs, which may be largely driven by tissue-specific TFs. eRNAs are involved in multiple cancer signaling pathways through putatively regulating their target genes, including clinically actionable genes and immune checkpoints. They may also affect drug response by within-pathway or cross-pathway means. We characterize the oncogenic potential and therapeutic liability of one eRNA, *NET1e*, supporting the clinical feasibility of eRNA-targeted therapy. We identify a panel of clinically relevant eRNAs and developed a user-friendly data portal. Our study reveals the transcriptional landscape and clinical utility of eRNAs in cancer.

## Abstract (web summary)

Enhancer RNA (eRNA) is a type of noncoding RNA transcribed from enhancer regions. Here, the authors investigate the transcriptional landscape of eRNA in cancer, incorporating pharmacogenomic data to identify potential target genes and therapeutic targets.

## Introduction

Enhancer is a distal regulatory DNA that enhances the transcription of a target gene by interacting with target gene promoter^1^. Traditionally considered to be DNA elements that nucleate transcription factor (TF) binding, enhancers were recently found to also transcribe noncoding RNAs, which are referred to as enhancer RNAs (eRNAs)^2^. Tens of thousands of eRNAs have been identified in human cells, many of which were shown to play important roles in transcriptional circuitry to mediate the activation of target genes^3^.

In human cancers, activation of oncogenes or oncogenic signaling pathways often converges to enhancer activation and production of eRNAs. For example, the activation of *ESR1* can globally increase eRNA transcription in breast cancer^4^. Oncogene-induced eRNAs can under certain circumstances directly promote tumorigenesis. For example, *KLK3e*, an androgen-induced eRNA regulating gene *KLK3*, can scaffold the androgen receptor (*AR*)-associated protein complex to control *AR*-dependent gene expression in prostate cancer^5^. Tumor suppressors can also induce eRNAs to contribute to tumor repression processes. For example, *TP53*-induced eRNAs were found to be involved in p53-dependent cell cycle arrest in multiple cancer cell lines^6^. Together these evidences reveal significant roles of eRNAs in tumorigenesis and suggest their clinical utility in eRNA-targeted therapy^7^.

The Encyclopedia of DNA Elements (ENCODE) project^8^, Functional Annotation of the Mammalian Genome (FANTOM) project^9^, and Roadmap Epigenomics project^10^ have annotated a large number of regulatory elements, including enhancers, while The Cancer Genome Atlas (TCGA) collected multi-omic data and clinical information in ~10,000 tumor samples^11^. In addition to patient samples, Cancer Cell Line Encyclopedia (CCLE)^12^ collected omics data in ~1000 cancer cell lines. Furthermore, Cancer Therapeutics Response Portal (CTRP)^13^, and Genomics of Drug Sensitivity in Cancer (GDSC)^14^ provided pharmacogenomics data from ~ 500 anticancer compounds across > 1000 cancer cell lines. These data resources provide unique opportunities to characterize the expression landscape, functions and drug response of eRNAs across different cancer types.

## Results

### Dynamic expression landscape of eRNAs in human cancers

**Fig. 1:**
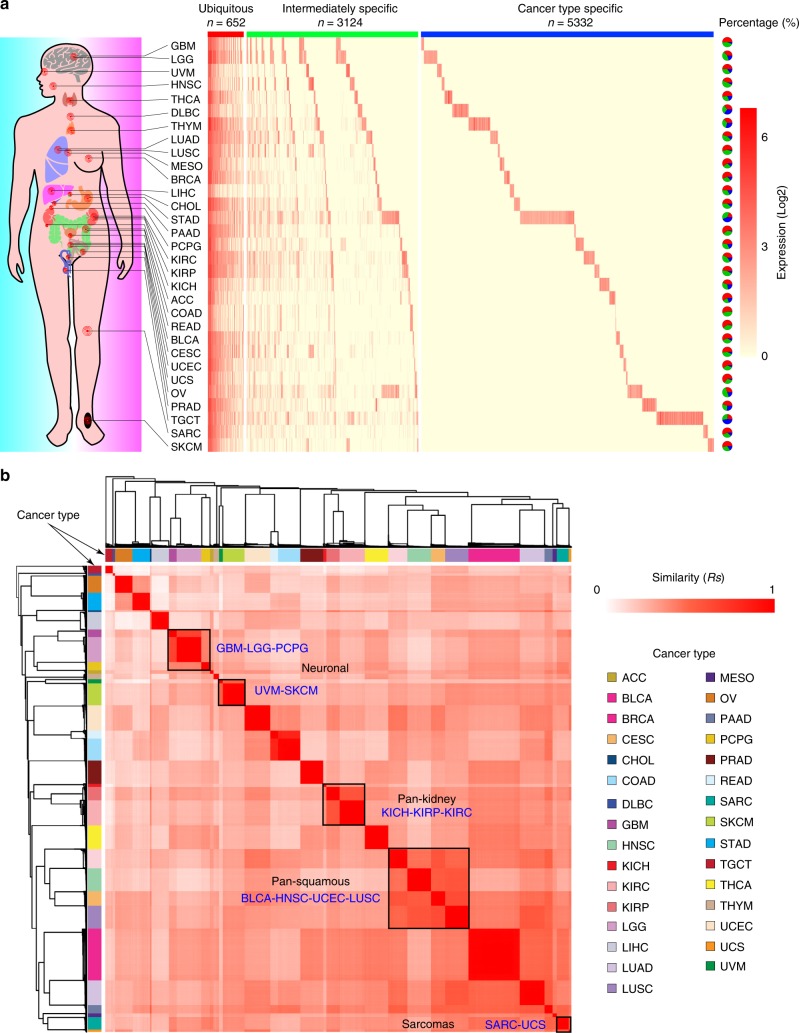
Expression landscape of eRNAs in human cancers. **a** Expression profile of eRNA in human cancers. Red, green and blue bars, respectively, denote ubiquitous, intermediately specific and cancer-type-specific eRNAs. Pie charts reflect the percentage of each category of eRNAs in each cancer type. Abbreviation of each cancer type is listed in Supplementary Data 1. Scale bar denotes the expression level of eRNAs (log2). Organ icons made by Freepik (https://www.freepik.com/). **b** Expression similarity among tumor samples. Color bar denotes each cancer type. Scale bar denotes the similarity of eRNA expression among TCGA samples

We obtained enhancer annotations from ENCODE, FANTOM, and Roadmap Epigenomics, and selected enhancers annotated in at least two datasets. Given the fact that eRNA transcription region could be wider than the enhancer ChIP-seq peaks^15^, we defined the ± 3 kb regions around the middle point of these annotated enhancers as potential eRNA-transcribing regions^16^. To avoid counting the transcriptional signal from known coding genes, we excluded eRNA regions that overlap with known coding genes and lncRNAs (with 1 kb extension from both transcription start site and transcription end site) (Supplementary Fig. 1A and Methods). To characterize the expression landscape of eRNAs across human cancers, we mapped TCGA RNA-seq reads to eRNA regions and defined those eRNAs with average expression value (reads per million, RPM) ≥1 as detectable eRNA for each cancer type (Supplementary Fig. 1A and Methods). This analysis identified a total of 9108 detectable eRNAs in human cancers (Fig. 1a and Supplementary Fig. 1B). The number of detectable eRNAs ranged from 457 in liver hepatocellular carcinoma (LIHC) to 2267 in stomach adenocarcinoma (STAD) (Supplementary Fig. 1B, Supplementary Data 1). We classified these detectable eRNAs into three groups: 652 ubiquitous eRNAs expressed in ≥10 cancer types, 3124 intermediately specific eRNAs that are expressed in 2–9 cancer types, and 5332 cancer-type-specific eRNAs that are expressed in only one cancer type (Fig. 1a). The ubiquitous eRNAs account for 20.0% of detectable eRNAs in STAD, but for 64.8% of eRNAs in uterine corpus endometrial carcinoma (UCEC). Interestingly, the ubiquitous eRNAs have higher expression levels than the intermediately specific eRNAs (Wilcoxon test *p*-value < 2.2 × 10^–16^) and the cancer-type-specific eRNAs (Wilcoxon test *p*-value < 2.2 × 10^–16^, Supplementary Fig. 1C). This phenomenon is reminiscent of features of protein-coding genes, among which the housekeeping genes are generally expressed at high levels as compared with tissue-specific genes^17^. Numbers of cancer-type-specific eRNAs showed a broad range, from 4 in colon adenocarcinoma (COAD) to 987 in STAD (Fig. 1a). We still observed cancer-type-specific pattern even with a much more stringent cutoff (RPM ≥ 5, Supplementary Fig. 1D), suggesting that the cancer-type-specific patterns of eRNA expression is not due to expression levels.

The expression similarity between any two tumor samples further showed a strong cancer-type-specific pattern, in that samples from the same cancer type clustered together (Fig. 1b). Furthermore, cancer types with similar histological features clustered at higher levels of hierarchy, such as pan-kidney cancers^18^ (kidney renal clear cell carcinoma [KIRC], kidney renal papillary cell carcinoma [KIRP] and kidney chromophobe [KICH]), pan-squamous cell carcinomas^19^ (bladder urothelial carcinoma [BLCA], head and neck squamous cell carcinoma [HNSC], cervical squamous cell carcinoma and endocervical adenocarcinoma [CESC] and lung squamous cell carcinoma [LUSC]), the sarcomas^20^ (sarcoma [SARC] and uterine carcinosarcoma [UCS]), and the neuronal cancers^20^ (glioblastoma multiforme [GBM], brain lower grade glioma [LGG], pheochromocytoma and paraganglioma [PCPG], skin cutaneous melanoma [SKCM], and uveal melanoma [UVM]). This cancer-type-specific pattern is further confirmed by t-Distributed Stochastic Neighbor Embedding (t-SNE) analysis (Supplementary Fig. 1E), suggesting that eRNAs may be powerful biomarkers with clinical utility in specific cancer types^7^.

### Analysis of transcription factor and eRNA relationship

**Fig. 2:**
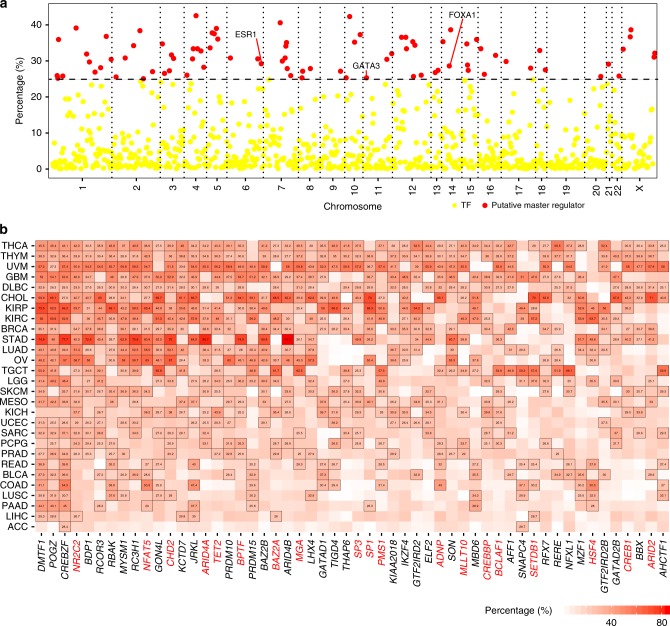
Putative regulation of eRNA biogenesis in cancer. **a** Putative regulators of eRNAs in BRCA. Each dot represents one transcription factor (TF). Red dots denote putative master regulators significantly correlated to ≥25% of individual eRNAs. Three well-known TFs (*ESR1*, *GATA3*, and *FOXA1*) are highlighted. *X*-axis is chromosome; *y*-axis is percentage of eRNAs correlated to each TF. **b** General putative master regulators in human cancers. *X*-axis is symbol of putative master regulator and *y*-axis is cancer type. General putative master regulators involved in genomic instability are marked by red. Number in cells denotes the percentage of eRNAs regulated by each master regulator. Scale bar denotes percentage of eRNA correlated with TFs (*x* axis) across cancer types (*y* axis)

TFs have been shown to mediate the biogenesis of eRNAs^2,8^; however, the global regulation of eRNAs is still unclear. Here, we collected human transcription factors (TFs) from JASPAR^21^, DBD^22^, AnimalTFDB^23^, and TF2DNA^24^, and calculated Spearman’s correlation between individual TF expression and individual eRNA expression in each cancer type. We defined TFs that show significant correlation (*Rs* ≥ 0.3; false discovery rate [FDR] <0.05) with an individual eRNA in a specific cancer type as its putative regulators, and further defined those putative regulators significantly correlated to ≥ 25% of individual eRNAs in a specific cancer type as putative master regulators. Taking breast invasive carcinoma (BRCA) as an example, we identified 84 putative master regulators, including three well-known regulators, *FOXA1*^25^ (highly correlated with 28.6% of all eRNAs in BRCA), *ESR1*^15^ (29.2%), and *GATA3*^26^ (25.3%, Fig. 2a and Supplementary Data 2). Applying this computational predictions, we have identified 845 putative master regulators across cancer types (Supplementary Fig. 2A and Supplementary Data 2). The majority of these putative master regulators (693/845, 82.0%) exhibits strong expression correlation with a large number of eRNAs in only one or a few cancer types (i.e., <5), suggesting that the TF-eRNA correlation is tissue-specific and may imply direct regulatory functions of these TFs in that cancer type (Supplementary Fig. 2B). For example, *OLIG2* is a TF highly expressed in brain and highly correlate with the expression of 33.5% of eRNAs in LGG, suggesting its potential importance in enhancer/eRNA control therein (Supplementary Fig. 2C). Our global analysis of TF-eRNA correlation indicates that cancer- and/or lineage-specific patterns of eRNAs can be largely mediated by lineage-specific TFs.

We further identified 54 general putative master regulators that play significant roles in ≥ 10 cancer types (Fig. 2b). We performed GO analyses and observed that these TFs are enriched in the functional categories related to transcriptional process (Supplementary Fig. 2D-2E). These general master regulators can be classified into 17 families base on Pfam annotation (https://pfam.xfam.org/), and they are significantly enriched in four families, including MDB, ARID, GTF2I, and MYB (FDR <0.05, Supplementary Fig. 2F). More importantly, we manually examined the functions of these TFs and found 35.2% (19/54) of them are associated with genomic instability (Fig. 2b). For example, *NR2C2*, which can mediate genomic rearrangements by a telomere-related pathway^27^, is highly correlated with eRNAs in 20 cancer types, ranging from 26.3% in PRAD to 53.7% in KIRP. *NFAT5*, which can induce genomic instability by regulating inflammation^28,29^, is highly correlated with eRNAs in 17 cancer types, ranging from 27.0% in READ to 63.4% in KIRP. These general master regulators are enriched in functions related to genomic instability, which provides a potential explanation to a previously observed positive correlation between somatic copy number alteration and enhancer activation in many cancer types^30^.

### Putative effects of eRNAs on signaling pathways

**Fig. 3:**
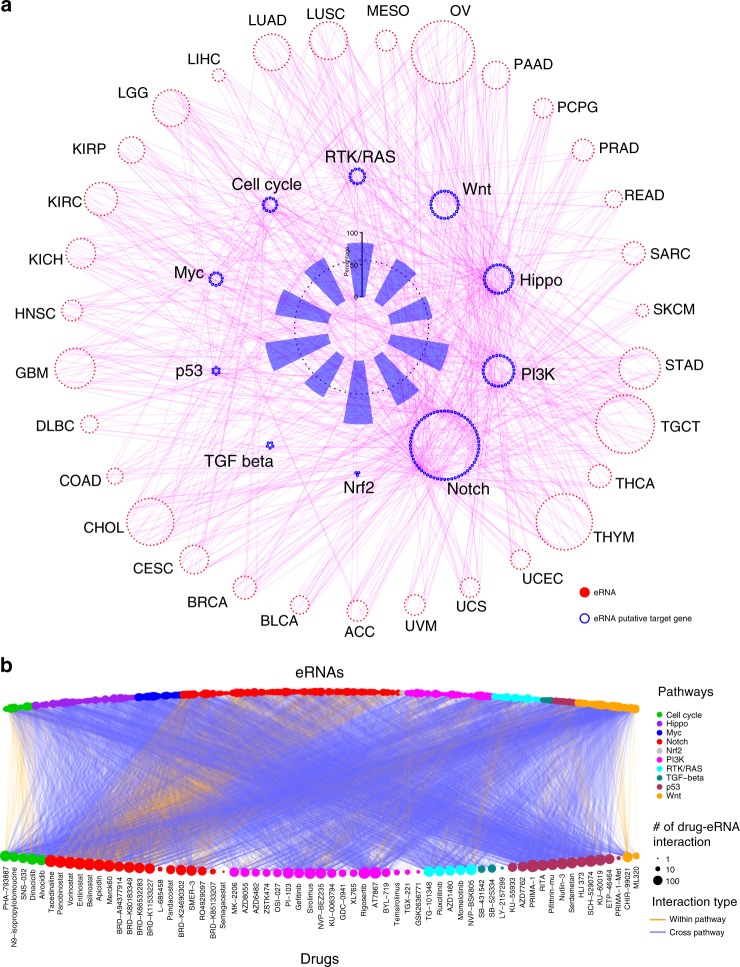
Putatively regulatory network and drug response of eRNAs on signaling pathways. **a** eRNA and putative target gene in 10 cancer signaling pathways. Red nodes in outer circle denote eRNAs that regulate genes in cancer signaling pathways across cancer types. Blue nodes in middle circle denote putative target gene across cancer signaling pathways. Blue bars in inner circle denote percentage of eRNA putative target genes across each pathway. Magenta links denote correlation between eRNAs and their putative target genes. **b** Association between eRNAs (top) and drugs (bottom) across different cancer signaling pathways. Orange and blue links, respectively, denote within- and cross-pathway relationships

It remains a challenge to establish the direct interaction between eRNA and its target genes. We built a global eRNA-gene regulatory network across cancer types based on the physical distance (≤ 1MB) and co-expression between individual eRNAs and their putative target genes (Spearman’s correlation *Rs* ≥ 0.3, FDR < 0.05, Supplementary Fig. 3A)^2^. We identified 11,593 (56.5% of all protein-coding genes) putative target genes that are significantly correlated with 88.8% (8086/9108) of eRNAs in at least one cancer type. High-throughput chromosome conformation capture (Hi-C) data can reveal the interaction between an enhancer and its target gene, while active enhancer usually produce eRNA^31–33^. Therefore, we investigated Hi-C interaction for all putative eRNA-gene connections across 20 tissues, and observed that more than 80% eRNA-gene connections are supported by significant Hi-C interactions in at least one tissue (Supplementary Fig. 3B). The proportion of Hi-C supported eRNA-gene connection is significant higher than the background of random pairs (permutation test, bootstrap = 10,000, *p* < 0.0001, Supplementary Fig. 3B). To explore the regulatory roles of eRNAs in cancer, we collected 229 genes involved in 10 cancer signaling pathways, including Myc, PI3K, and p53 pathways^34^ (Supplementary Data 3). The majority (185/229, 80.8%) of these genes are correlated with eRNAs in at least one cancer type (Fig. 3a and Supplementary Data 3). For example, all six genes in the p53 pathway (i.e., *TP53*, *MDM2*, *MDM4*, *ATM*, *CHEK2*, and *RPS6KA3*) were found to correlate with eRNAs in at least one cancer type (Supplementary Fig. 3C and Supplementary Data 3). In support of this, most eRNA-gene associations in pathways (91.9%, 170/185) were found to form chromatin interaction by Hi-C (Supplementary Fig. 3D and 3E), including *MAML2*-associated eRNA (hereafter we will refer to eRNAs based on their associated, putative target gene, i.e., *MAML2e*, ENSR00000043746) and *MAML2*, *CDK6*-associated eRNA (*CDK6e*, ENSR00000215101) and *CDK6*, and *TCF7L2*-associated eRNA (*TCF7L2e*, ENSR00000033597*)* and *TCF7L2* (Supplementary Fig. 3F). Our results suggested important roles played by eRNAs in regulating various cancer signaling pathways.

To further understand the meaningful contributions of eRNAs in cancer signaling pathways on drug response, we calculated eRNA expression levels across ~1000 cancer cell lines from the Cancer Cell Line Encyclopedia (CCLE), and then analyzed Spearman’s correlation between eRNA expression levels and drug sensitivity of these cells (Area Under Curve [AUC]), which is available from the Cancer Therapeutics Response Portal (CTRP). We identified 512 eRNAs in all 10 cancer signaling pathways, the expression of which displayed high correlation with 63 anticancer drugs (FDR < 0.05^35^, Fig. 3b and Supplementary Fig. 3G), suggesting significant roles of eRNAs in the response to anticancer drugs. For example, 217 eRNAs are highly correlated with belinostat, a drug that targets the Notch pathway. Among these, 32.7% (71/217) of their putative target genes are within the Notch pathway (Supplementary Fig. 3H), such as *PSEN2*-associated eRNA (*PSEN2e*, ENSR00000257043)*, RBX1*-associated eRNA (*PBX1e*, ENSR00000257043), and *NOTCH4* associated eRNA (*NOTCH4e*, ENSR00000320261). More interestingly, the putative target genes of the remaining eRNAs (146/217, 67.4%) are in cross-pathways, such as *MDM2*-associated eRNA (*MDM2e*, ENSR00000053727) in the p53 pathway, *CDK6*-associated eRNA (*CDK6e*, ENSR00000215101) in the cell cycle pathway, and *RNF43*-associated eRNA (*RNF43e*, ENSR00000096250) in the Wnt pathway (Supplementary Fig. 3H). We further confirmed this eRNA-drug connection using another drug database, Genomics of Drug Sensitivity in Cancer (GDSC), and observed some similar pattern (Supplementary Fig. 3I and 3J). Indeed, belinostat treatment could alter the expression of 46 eRNAs (35.7%) within the target pathway and 83 eRNAs (64.3%) in a cross-pathway in A549 cells (Supplementary Fig. 3K). Taken together, our results suggest a strong association between eRNAs and anticancer drugs, either within the target pathway or through a cross-pathway. It will an important future direction to examine the molecular basis of eRNA-gene-drug correlation, and potential roles eRNAs played in modulating cancer cell drug response.

### Putatively regulation of eRNAs on CAGs and ICs

**Fig. 4:**
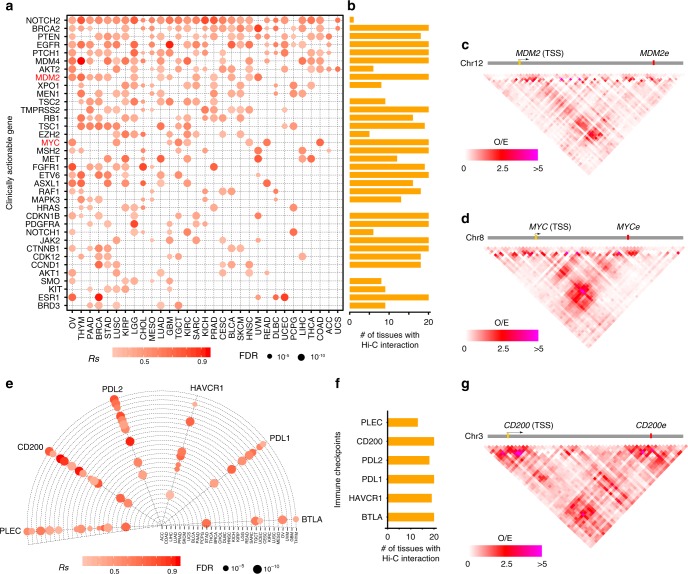
Putatively regulatory effects of eRNAs on clinically actionable genes and immune checkpoints. **a** Correlation between eRNAs and clinically actionable genes in human cancers. Red dots denote significantly correlation. *X*-axis is cancer type and *y*-axis is symbol of gene. **b** Number of tissues with Hi-C interactions between clinically actionable genes and their eRNAs. **c** Hi-C interaction between *MDM2* and *MDM2e* in blade. **d** Hi-C interaction between *MYC* and *MYCe* in lung. **e** Correlation between eRNA and cancer immune checkpoints in human cancers. **f** Number of tissues with remarkable Hi-C interaction between cancer immune checkpoints and their eRNA. **g** Hi-C interaction between *CD200* and *CD200e* in ovary. Scale bars denote spearman correlation (*Rs*) in (**a**) and (**e**), and Hi-C O/E value in (**b**), (**c**), and (**g**), respectively

Based on the finding that eRNAs were tightly associated with cancer signaling pathways and drug-associated pathways, we further asked if eRNAs were directly linked to cancer therapy. We collected 135 clinically actionable genes (CAGs), and observed that 107 of them (79.3%) were correlated to eRNAs in at least one cancer type (distance ≤ 1MB, Spearman’s correlation *Rs* ≥ 0.3 and FDR < 0.05, Supplementary Data 4). Among these, 36 clinically actionable genes are correlated with eRNAs in at least five cancer types (Fig. 4a), suggesting that these genes are potentially regulated by eRNAs in multiple cancers. Increased numbers of samples enhance the ability to detect and analyze molecular data. In particular, the pan-cancer analysis will help to identify master events that play a critical functional role in different tumor contexts^36–38^. Furthermore, 91.7% of these correlations (33/36) could be supported by Hi-C interactions in at least one tissue, which further support the potential regulatory roles of eRNAs on clinically actionable genes (Fig. 4b and Supplementary Fig. 4A). For example, *MDM2-* and *MDM2*-associated eRNA *(MDM2e*, ENSR00000053727*)* are positively correlated in 12 cancer types, and Hi-C data supports their chromatin interaction in 20 tissues (Fig. 4c and Supplementary Fig. 4A). *MYC*- and *MYC*-associated eRNA (*MYCe*, ENSR00000333355) are positively correlated in six cancer types, and Hi-C data supports their interaction in all 20 tissues (Fig. 4d and Supplementary Fig. 4A).

We further investigated the relationship between individual eRNAs and immune checkpoints (ICs, Supplementary Data 5), and observed six checkpoints were correlated with eRNAs in at least five cancer types (Fig. 4e). All these putative eRNA-checkpoints interactions again were supported by Hi-C data in at least one tissue (Fig. 4f and Supplementary Fig. 4B). For example, *CD200-* and *CD200*-associated eRNA (*CD200e*, ENSR00000156542) are positively correlated in 12 cancer types, and Hi-C data supports the interactions in all 20 tissues (Fig. 4g and Supplementary Fig. 4B). Taken together, our analysis showed putative interactions between eRNAs and clinically actionable genes and/or immune checkpoints, suggesting the potentially clinical utility of eRNAs in cancer therapy.

### Characterizing the functional roles of eRNA in cancer

**Fig. 5:**
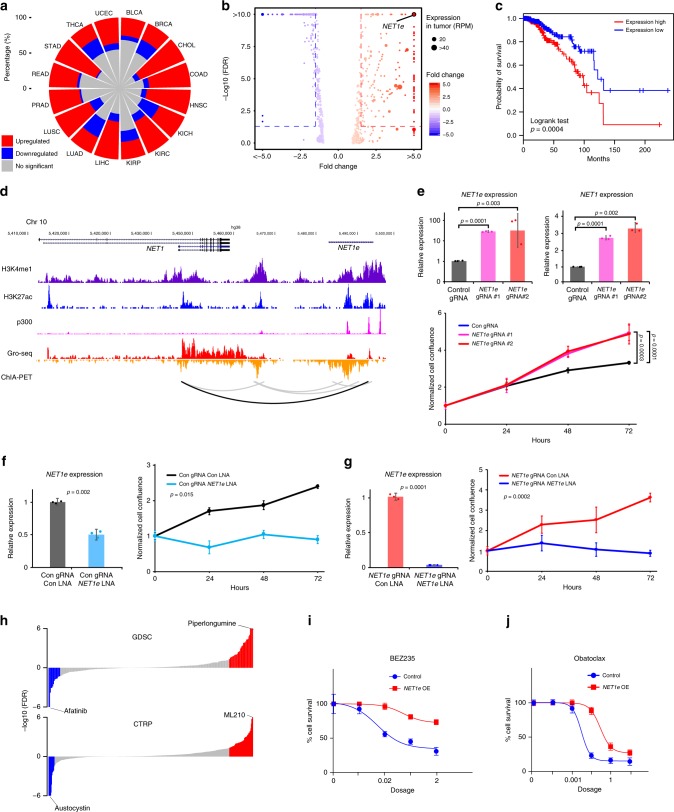
Functional characterization of *NET1e* in cancer. **a** Expression alterations of eRNAs in human cancers. Red, blue and gray, respectively, denote percentage of upregulated, downregulated and no significant expression of eRNAs in each cancer type. **b** Differentially expressed eRNAs in BRCA. Scale bar denotes the fold change between tumor and normal samples. **c** Survival curves for *NET1e* in BRCA (top quarter in red vs. bottom quarter in blue). **d** Epigenetic features and expression of *NET1e* and *NET1* region in MCF7. **e** Overexpression of *NET1e* in MCF7 and its functional consequences. Overexpression of *NET1e* by CRISPR/dCas9-SAM (top left) and *NET1* (top right), and cell growth assay with overexpression of *NET1e* in MCF7 (bottom). **f** Knockdown *NET1e* by LNA (left) and cell growth assay (right) in MCF7. **g** Knockdown *NET1e* by LNA after activation (left) and cell growth assay (right) in MCF7. **h** *NET1e-*associated drugs in GDSC and CTRP database. **i** Response curve of compound BEZ235 in *NET1e* overexpressed cells and control. **j** Response curve of compound Obatoclax in *NET1e* overexpressed cells and control. Error bars in (**e**), (**f**), (**g**), (**i**), and (**j**) indicate mean ± SD

To further characterize the functional roles of eRNAs in cancer, we examined the differentially expressed eRNAs (|fold change| >1.5 and FDR <0.05) across 16 cancer types with ≥5 tumor-normal paired samples (Supplementary Data 6). Overall, there were more upregulated eRNAs in tumor samples, ranging from 22.0% in thyroid carcinoma (THCA) to 68.9% in cholangiocarcinoma (CHOL), with a median of 42.2%. The downregulated eRNAs ranged from 1.9% in STAD to 27.9% in KICH, with a median of 9.9% (Fig. 5a). Taking BRCA as an example, we identified 208 upregulated eRNAs and 166 downregulated eRNAs (Fig. 5b). Among these, one eRNA located ~ 90 kb downstream of the oncogene *NET1*^39^, which we referred to as *NET1-*associated eRNA (*NET1e*, ENSR00000023843*)*, showed the largest expression alteration between tumor and normal samples (fold change = 5.8, FDR = 3.7 × 10^–13^, Fig. 5b and Supplementary Fig. 5A). *NET1e* exhibited much higher expression levels in BRCA, including all subtypes (Supplementary Fig. 5B). High level of *NET1e* was associated with worse survival (log-rank test *p*-value = 0.0004, Fig. 5c). Of interest, *NET1* gene itself is not associated with the breast cancer patient’s survival (Supplementary Fig. 5C), suggesting that *NET1e* may be a predictor irrelevant to *NET1* in breast cancer patients. *NET1e* was highly correlated with *NET1* across all BRCA subtypes (Fisher’s transformation, *Rs’* = 0.45, *p*′ = 1.58 × 10^–4^), including the basal subtype (Spearman’s correlation, *Rs* = 0.53, *p* = 1.95 × 10^–11^, Supplementary Fig. 5D). We further examined *NET1e* signaling in MCF7, a breast cancer cell line (Fig. 5d). This region harbors classical enhancer features, such as the enrichment of histone H3K4me1 modification; it also exhibits strong enrichment of active enhancer markers such as histone H3K27ac modification and binding of transcription co-factor p300 (Fig. 5d). There are multiple p300 binding peaks densely distributed in the *NET1e* region, indicating it might be a potential super-enhancer in MCF7. *NET1e* transcription was also detected by GRO-seq data in MCF7 cells (Fig. 5d). Furthermore, we observed a chromatin interaction between *NET1e* and *NET1* by RNA Pol II ChIA-PET^21^, suggesting a direct interaction for regulation.

To further characterize *NET1e*, we applied CRISPR activation (CRISPR/dCas9-SAM)^40^ to epigenetically induce *NET1e* expression in MCF7 cells (Fig. 5e). We successfully achieved >30-fold *NET1e* upregulation by two different sgRNAs, which interestingly led to strong upregulation of *NET1* mRNA (Fig. 5e). Consistent with the role of *NET1* as a breast cancer oncogene^39^, CRISPR-SAM induction of *NET1e* increased cell proliferation significantly (Fig. 5e). To delineate a role of the eRNA per se, we designed three locked nucleic acid GapmeR (LNAs) to knockdown *NET1e*. With efficient reduction of *NET1e* expression, we found that cell proliferation was significantly reduced in both MCF7 cells (Fig. 5f) and MCF7 cells with CRISPR-SAM treatment (Fig. 5g). These data, together with their chromatin looping (Fig. 5d), suggested that *NET1e* contributes to breast cancer progression via upregulation of the important breast cancer oncogene *NET1*. In addition, knockdown of *NET1e* did not significantly impact cell proliferation in non-breast cancer cell lines, including MCF10A and Hela, in which *NET1e* shows low expression level (Supplementary Fig. 5E and 5F), suggesting a specific effect of *NET1e* in breast cancer growth. It supports a minimal off-target effects/toxicity of *NET1e* LNA and the potential to target cancer-specific eRNAs for effective treatment. More importantly, expression of *NET1e* is negatively correlated (sensitive, Spearman’s correlation, FDR <0.05) with 14 and 15 compounds response (AUC) while positively correlated (resistance, Spearman’s correlation, FDR < 0.05) with 56 and 31 compounds response (AUC) in CTRP and GDSC, respectively, which suggested that altered expression of *NET1e* could influence response to these drugs (Fig. 5h and Supplementary Data 7). Indeed, in situ overexpression of *NET1e* led to the resistance of MCF7 cells to a PI3K inhibitor, BEZ235 (Fig. 5i), and a BCL-2 Inhibitor, Obatoclax (Fig. 5j) in MCF7 cells. We also examined the effects for the other three drugs (CHIR-99021, BX-795, and (5Z)-7-Oxozeaenol) and observed a similar trend but not statistically significant. Cells showed strong growth inhibition when we knocked down *NET1e* in MCF7 (Fig. 5f, g), therefore we could not test drug response in *NET1e* KD cells. Of interest, *NET1* is not significantly correlated with BEZ235 (FDR = 0.15) and obatoclax (FDR = 0.80). Taken together, these results revealed that *NET1e* is an oncogenic eRNA in BRCA and may be a promising target for eRNA therapy.

### Identification of clinically relevant eRNAs

**Fig. 6:**
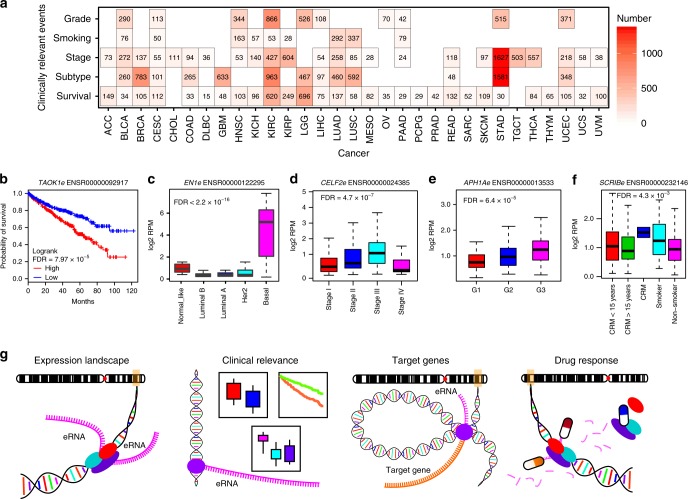
Clinically relevant eRNAs and the eRNA data portal in cancer (eRic). **a** Number of clinically relevant eRNAs across different cancer types. For survival analyses, we use cox model to analyze the relationship between eRNA expression and survival time, and considered FDR < 0.05 as significant. For other clinical relevance, we used Student’s *t* test to test the difference between two groups and analysis of variance (ANOVA) to test the difference among multiple groups, and considered FDR < 0.05 as significant. Scale bar denotes number of clinically relevant eRNAs. **b**–**f** Examples of clinically relevant eRNAs for patient survival time (**b**), cancer subtype (**c**), tumor stage (**d**), tumor grade (**e**), and patient smoking history (**f**). The boxes in (**c**–**f**) show the median ± 1 quartile, with whiskers extending from the hinge to the smallest or largest value within 1.5 interquartile range from the box boundaries. **g** The four modules in eRic: expression landscape, clinical relevance, putative target genes, and drug response

Clinical relevance is used to define cancer-related clinical features, including association with survival, differential expression among subtypes, stages, grade, and different groups of smoking history^41–43^. To further investigate the clinical utility of eRNAs, we identified 5715 clinically relevant eRNAs (i.e., associated with clinical relevance) that account for 62.7% (5715/9108) of the total detectable eRNAs in cancers (Fig. 6a and Supplementary Data 8). For example, *TAOK1*-associated eRNA (*TAOK1e*, ENSR00000092917), which putatively targets the Hippo signaling pathway gene *TAOK1*^44^, is associated with overall survival in KIRC (Fig. 6b, log-rank test, FDR = 7.97 × 10^–5^); *EN1*-associated eRNA (*EN1e*, ENSR00000122295), which putatively targets the BRCA-basal marker gene *EN1*^45^, is highly expressed in the BRCA-basal subtype (Fig. 6c, Analysis of variance [ANOVA], FDR < 2.2 × 10^–16^); *CELF2*-associated eRNA (*CELF2e*, ENSR00000024385), which putatively targets the tumor suppressor gene *CELF2*^46^, is highly expressed in stage III STAD (Fig. 6d, ANOVA, FDR = 4.7 × 10^–7^); *APH1A*-associated eRNA (*APH1Ae*, ENSR00000013533), which putatively targets the oncogene *APH1A*^47^, is highly expressed in grade-3 LIHC (Fig. 6e, ANOVA, FDR = 6.4 × 10^–5^); and *SCRIB*-associated eRNA (*SCRIBe*, ENSR00000232146), which putatively targets the oncogene *SCRIB*^48^, is differentially expressed among patients with LUAD according to different categories of smoking history (Fig. 6f, ANOVA, FDR < 4.3 × 10^–3^). These results suggest that appreciable levels of eRNAs are clinically valuable.

### A comprehensive data resource to explore eRNAs in cancer

We developed a user-friendly data portal, eRNA in cancer (eRic) (https://hanlab.uth.edu/eRic), to facilitate broad access to these data by the biomedical community. eRic includes four modules: expression, clinical relevance, target genes and drug response (Fig. 6g). In the eRNA-expression module, users can explore the expression of eRNA across TCGA cancer types and samples and the eRNA location by Ensembl ID or genomic location. The clinical relevance module aims to help users identify clinically relevant eRNAs, including those that showed differentially expressed patterns between tumor and normal samples among different groups of cancer subtypes, stages, and grades and different categories of patient smoking history, and in association with patient survival times. The target genes module allows users to identify eRNA target genes (Supplementary Fig. 6A). We also integrated the drug response data from GDSC and CTRP, which allows users to investigate whether an eRNA shows sensitivity or resistance to drugs (Supplementary Fig. 6B and 6C). In addition, eRic provides a download module, which allows users to download the expression, clinical relevance, targeted genes, and drug response data. This valuable resource will be of significant interest to the research community^49^.

## Discussion

eRNA are increasingly realized to play important roles in the regulation of gene transcriptional circuitry in human cancers. We developed a computational pipeline to reveal the global expression landscape of eRNAs across multiple cancer types. By integrating multi-omics data from TCGA, CCLE, ENCODE, FANTOM, Roadmap Epigenomics, and 4D Nucleome projects, as well as pharmacogenomics datasets from GDSC and CTRP, we have revealed novel insights on the expression landscape and clinical utility of eRNAs in cancer (Supplementary Fig. 7 and Supplementary Table 1). We demonstrated a strong cancer-type-specific expression pattern of many eRNAs, suggesting that eRNAs may be powerful diagnostic and/or prognostic markers in cancer therapy. The cancer-type-specific pattern is aligned well with the previous studies characterizing the regulatory elements (e.g., enhancers) based on ATAC-seq^50^, as well as activated enhancers^30^. The sequencing depth may still cause some batch effects. For those cancer types with 75/76 bp pair-end reads, including OV, STAD, and GBM, only STAD showed the relative more eRNAs. In contrast, COAD, READ, and UCEC with 76 bp single-end reads have relative fewer eRNAs. The majority (25 out of 31) of cancer types with 48/50 bp pair-end reads showed vast difference in detectable eRNAs ranging from 457 in LIHC to 1790 in TGCT (Supplementary Data 1). The number of eRNAs is not correlated with the sequencing depth in these cancer types (*Rs* = 0.02, *p* = 0.93). These results suggested that the tissue-specific pattern of eRNAs is robust. We identified a series of transcription factors as the potential regulators for eRNA biogenesis, which greatly expanded our knowledge about eRNA biogenesis. Interestingly, we observed that the general putative master regulators of eRNAs, including *NR2C2* and *NFAT5*, displayed an intriguing enrichment of functions in modulating genomic instability, suggesting a potential mechanistic link between eRNA expression/biogenesis and genome instability.

Integrative analysis showed that more than 80% genes in the canonical cancer signaling pathways are highly correlated with specific eRNAs in at least one cancer type, suggesting potentially important regulatory roles of eRNA in cancer. Due to the lack of Hi-C data in large number of tumor tissues, we can only confirm the eRNA-gene connections support by Hi-C interaction in at least one normal tissue. It will be more appropriate to use the Hi-C data in matched samples to confirm the regulatory roles of eRNAs. We also observed associations between eRNAs and anticancer drugs, either within the target pathway or through a cross-pathway. Furthermore, many clinically actionable genes and immune checkpoints were putatively regulated by eRNAs, emphasizing the clinical utility of eRNA in anticancer treatment. Nevertheless, our integrative analysis demonstrated the putative regulatory roles of eRNAs, and further experiments are necessary to confirm their regulatory roles.

We demonstrated the functional importance of an individual eRNA, *NET1e*, which is highly expressed in breast cancer. CRISPR activation of *NET1e* accelerated cell growth in MCF7, suggesting its oncogenic effect in cancer cell lines, while *NET1e* LNA specifically decreased cell proliferation in MCF7, and has shown limited or no off-target effects and toxicity. More importantly, in situ overexpression of *NET1e* will lead to drug resistance to BEZ235 and Obatoclax in MCF7 cells. To our knowledge, this is the first evidence showing that eRNA could affect drug response in cancer. Taken together, our results suggest the promising clinical importance of *NET1e*.

RNA-target drugs are now becoming a major new branch of pharmaceuticals. For example, US FDA has approved the first siRNA drug in 2018 (i.e., to use Patisiran infusion for the treatment of peripheral nerve disease like polyneuropathy), and there are extensive ongoing efforts to target disease-relevant RNAs in the pharmaceutical industry. We identified an appreciable number of clinically relevant eRNAs and further demonstrated their clinical utility in diagnostic and/or eRNA-targeted therapy. To facilitate utilization of the expression landscape and clinical relevance of eRNAs by the broad biomedical community, we have built a data portal, eRic, offering a comprehensive resource for further investigation of eRNA expression landscape, clinical relevance, target genes, functions in tumorigenesis or response to anticancer drugs. This is the first data portal in eRNA field and will be a valuable resource for further investigation of cancer therapy that targets eRNAs. In particular, the related data will help the researchers to identify key eRNAs in cancer patients, and to select the appropriate cancer cell lines for their functional investigations.

## Methods

### Data collection

We downloaded RNA-seq BAM files, clinical features and the mRNA expression matrix from TCGA data portal (https://portal.gdc.cancer.gov/)^11^. GRO-seq data and ChIP-seq for MCF7 were collected from our previous paper^4^. ChIA-PET data were obtained from WASHU EpiGenome Browser (https://epigenomegateway.wustl.edu/)^21^. RNA-seq data of cancer cell lines were downloaded from the Cancer Cell Line Encyclopedia (https://portals.broadinstitute.org/ccle/about)^46^. Drug sensitivity datasets were downloaded from GDSC^14^ and CTRP^51^. The Hi-C interactions across 20 human tissues were downloaded from http://promoter.bx.psu.edu/public/HiCPlus/matrix/^52^. Clinically actionable genes were collected from previous literatures^37,38,53^, and cancer immune checkpoints were collected from a previous literature^54^. RNA-seq data for A549 treated by belinostat (GSE96649) was downloaded from Gene Expression Omnibus (https://www.ncbi.nlm.nih.gov/geo/), and processed by Hisat2 software^55^ and SAMtools toolkit^56^.

### Quantification of eRNA expression

Annotation of enhancers were collected from Ensembl (https://useast.ensembl.org/)^57^, FANTOM (http://fantom.gsc.riken.jp/index.html)^9^, and Roadmap Epigenomics (http://www.roadmapepigenomics.org/)^10^. The annotation from ENCODE and Roadmap considered H3K4me1 and H3K27ac marks^57^, and annotation from FANTOM considered CAGE marks. We combined all three datasets and used those enhancers annotated in at least two datasets. Annotation of protein-coding genes was collected from GENECODE^58^ and UCSC Genome Browser^59^ (hg38). We used the ± 3 kb of the middle loci of enhancer to define eRNA region^16^. We also filtered out those eRNA regions that overlapped with known coding regions and lncRNAs (with 1 kb extension from both transcription start site and transcription end site). In particular, the ~ 500 bp (uaRNA) region was also excluded from our analysis. We also excluded all blacklist regions, including rRNA repeats.

We downloaded RNA-seq BAM files from TCGA data portal (https://portal.gdc.cancer.gov/)^11^. The RNA-seq raw data were processed by TCGA consortium as described on the official website (https://docs.gdc.cancer.gov/Data/Bioinformatics_Pipelines/Expression_mRNA_Pipeline/). We downloaded BAM files for our downstream analysis. We mapped RNA-seq data to these eRNA regions and calculated the expression level as RPM^60^ for each eRNA in each sample. We normalized eRNA expression by reads per million. We averaged all RPMs annotated to the eRNA from all samples in a cancer type, and defined those eRNAs with average expression level (RPM) ≥1 as a detectable eRNA. We converted different genomic versions of the human genome by liftover^59^. We present t-SNE analysis using R package Rtsne^61^. Our method may only detect a subset of polyadenylated eRNAs at their steady state since TCGA and CCLE only included the poly(A) RNA-seq. Our method may not distinguish functional eRNAs from those may just be the side effect of active enhancer.

### Biogenesis of eRNAs

We collected TFs from multiple TF resources, including JASPAR (http://jaspar.genereg.net/)^21^, DBD (http://www.transcriptionfactor.org/)^22^, AnimalTFDB (http://bioinfo.life.hust.edu.cn/AnimalTFDB/)^23^, and TF2DNA (http://www.fiserlab.org/tf2dna_db/)^24^. We identified putative regulators of eRNAs based on the co-expression between individual eRNA and each TF in a given cancer type, and considered Spearman’s correlation *Rs* ≥ 0.3 and FDR < 0.05 as significant. For each cancer type, TFs that significantly correlated with more than 25% of the detectable eRNAs were defined as master regulators. Master regulators that exist in more than 10 cancer types were defined as general master regulators. The functional enrichment analyses of these general master regulator were performed by DAVID^62^ and GSEA^63^.

### eRNA putative target genes and drugs

We identified eRNA putative target genes based on close distance (≤ 1MB) and co-expression (Spearman’s correlation *Rs* ≥ 0.3 and FDR < 0.05) between individual eRNAs and their putative target genes in each cancer type^58^. We filtered out eRNAs located in the intronic regions of target genes for correlation analysis. We collected 229 genes associated with 10 cancer signaling pathways^34^: p53, PI3K, Myc, RTK/RAS, cell cycle, Wnt, TGF beta, Nrf2, Notch, and Hippo. Due to the lack of Hi-C data in large number of tumor tissue, we used 20 Hi-C data from normal tissues^52^ to confirm the putative eRNA-gene connections. Hi-C interaction was evaluated by O/E value, which is calculated as observed value (estimated with normalized mapped reads) divided by expected value (estimated with a genome-wide model of interaction probability over the genomic distance)^64^. We also estimated the Hi-C interactions based on random eRNA-gene pairs throughout genome as background, and performed permutation test (bootstrap = 10,000) to compare with eRNA-gene pairs with the background of random pairs. For those eRNAs identified in TCGA, we examined their expression across ~1000 cancer cell lines in CCLE. GDSC and CTRP collected drug response data across >1000 cancer cell lines. We used matched cell lines to calculate the Spearman’s correlation between eRNA expression in CCLE and drug response value (AUC) of more than 500 anticancer drugs from CTRP and GDSC, and defined FDR < 0.05 as significant^35^.

### Clinically relevant eRNAs

We used the Student’s *t* test to assess the statistical difference between tumor and paired normal samples and defined significantly aberrant expression as |fold change| > 1.5 and FDR < 0.05. We used Student’s *t* test for two groups and analysis of variance (ANOVA) for multiple groups to assess the statistical difference of patient smoking history and cancer subtype, stage, and grade (FDR <0.05). Only groups with ≥ 5 samples were included in these analyses. We used the univariate Cox model or log-rank test to assess whether eRNA expression was associated with the overall survival times of cancer patients and considered FDR < 0.05 as significant.

### Lentivirus generation

A mixture of 3 μg of psPAX2, 1 μg of pMD2.G, and 4 μg of target sgRNA vector was transfected into 293T cells using Lipofectamine 2000 (Life Technologies). After 16 h, the media was changed, and the supernatants were collected at 48 and 72 h posttransfection for two independent infections. The collected supernatants were filtered using 0.45 μm syringe filter (Fisher) and used to infect MCF7 cells after being mixed with polybrene (final concentration of 8 μg ml^−1^, Sigma). Target cells were incubated in complete media with an equal amount of lentiviral particle-containing media for 24 h for each infection. After the second infection, the cells were selected over at least one week with selection markers to achieve a stable line.

### Cell culture and transfection

We originally purchased MCF7 and MCF10A cells from American Type Culture Collection. We maintained the MCF7 and Hela cells in Dulbecco’s modified Eagle’s medium (DMEM) (Corning) media, supplemented with 10% fetal bovine solution (FBS) (GenDEPOT) and maintained the MCF10A cells in DMEM/F-12 (Corning) supplemented with 5% horse serum, 20 ng ml^−1^ EGF, 0.5 mg ml^−1^ hydrocortisone, 100 ng ml^−1^ cholera toxin, 10 μg ml^−1^ insulin in a 5% CO_2_ incubator at 37 °C^4,65^. Transfection of LNA GapmeRs (Qiagen) into the cells was carried out using Lipofectamine 2000 (Life Technologies) according to the manufacturer’s protocol and at a final concentration of 60 nM. For *NET1e* eRNA knockdown, a mixture of *NET1e* LNA 1, 2, and 3 was transfected into the cell. The sequence information for LNA is described in Supplementary Table 2.

### CRISPR/dCas9-SAM

We followed the experimental procedures in Konermann et al.^40^. In brief, we generated the MCF7 stable cell line expressing dCAS9-VP64-Blast and Lenti MS2-p65-HSF1-Hygro using lentivirus. Infected cells were selected in DMEM supplemented with 10% FBS, 300 μg ml^−1^ Hygromycin, and 5 μg ml^−1^ Blasticidin. After 1 week of selection, we infected the stable cells with the lentiviral particle expressing sgRNA and selected the infected cells in DMEM with 10% FBS, 300 μg ml^−1^ Hygromycin, 5 μg ml^−1^ Blasticidin, and 300 μg ml^−1^ Zeocine for 1 additional week. All plasmids for CRISPR/dCAS9-SAM were purchased from Addgene (#61425, 61426, and 61427). The target gRNA sequence was chosen using http://crispr.mit.edu/. Target gRNA sequences were cloned into plasmid 61427 (Supplementary Table 2).

### qRT-PCR for eRNA expression

RNA was extracted from cells using Quick RNA-miniprep (Zymo Research) and the RNA was reverse-transcribed using SuperScript® III Reverse Transcriptase with random hexamer (Invitrogen) or qScript XLT cDNA SuperMix (QuantaBio). We performed qRT-PCR in QuantStudio 3 qPCR systems (Applied Biosystems, Thermo Fisher) using 2X Ssoadvanced Universal Sybr Green Supermix (Bio-Rad). We used glyceraldehyde-3-phosphate dehydrogenase for normalization. We used a two-tailed Student’s *t* test to obtain the *p*-values. The sequences of qPCR primers are provided in Supplementary Table 2. All RT-qPCRs were performed with at least biological duplicates. Each biological replicate has three technical repeats.

### Cell growth assay

Cells were trypsinized and plated at 3000 cells per well in a 96-well plate (Corning). Photos of each well were taken every 24 h using Incucyte Live Cell Imager (Essen Bioscience), and cell confluence was measured by Incucyte Software (Essen Bioscience) for 72 h. To normalize the confluences, the values for each time point were divided by the mean value at 0 h. In order to test the effects of *NET1e* to drug sensitivity in MCF7, we examined the half maximal inhibitory concentration (IC50) for PI3K inhibitor (BEZ235), BCL-2 Inhibitor (Obatoclax) in MCF7 Crispr/SAM control and *NET1e* using Incucyte live cell imager.

### Data portal

We constructed the data portal based on Rscript and JavaScript. The expression profile, clinical relevance, putative target genes, Hi-C data, and drug responses of eRNAs are available on the data portal (https://hanlab.uth.edu/eRic/).

### Reporting summary

Further information on research design is available in the Nature Research Reporting Summary linked to this article.

## Supplementary information

Supplementary Information

Description of Additional Supplementary Files

Supplementary Data 1

Supplementary Data 2

Supplementary Data 3

Supplementary Data 4

Supplementary Data 5

Supplementary Data 6

Supplementary Data 7

Supplementary Data 8

Reporting Summary

## Data Availability

All accession codes, unique identifiers, or web links for publicly available datasets are described in the paper. All data supporting the findings of the current study are listed in Supplementary Data 1–8, Supplementary Fig. 7, and our online data portal (https://hanlab.uth.edu/eRic/).

## Code availability

All codes are available upon reasonable request.
