# Supplementary material for: Transcriptional landscape and clinical utility of enhancer RNAs for eRNA-targeted therapy in cancer
Source: Nat Commun. 2019 Oct 8;10:4562. doi: 10.1038/s41467-019-12543-5 (PMC6783481; doi:10.1038/s41467-019-12543-5)
Supplement: Supplementary file 1 — Supplementary Information [file 41467_2019_12543_MOESM1_ESM.pdf]

Supplementary Figure 1

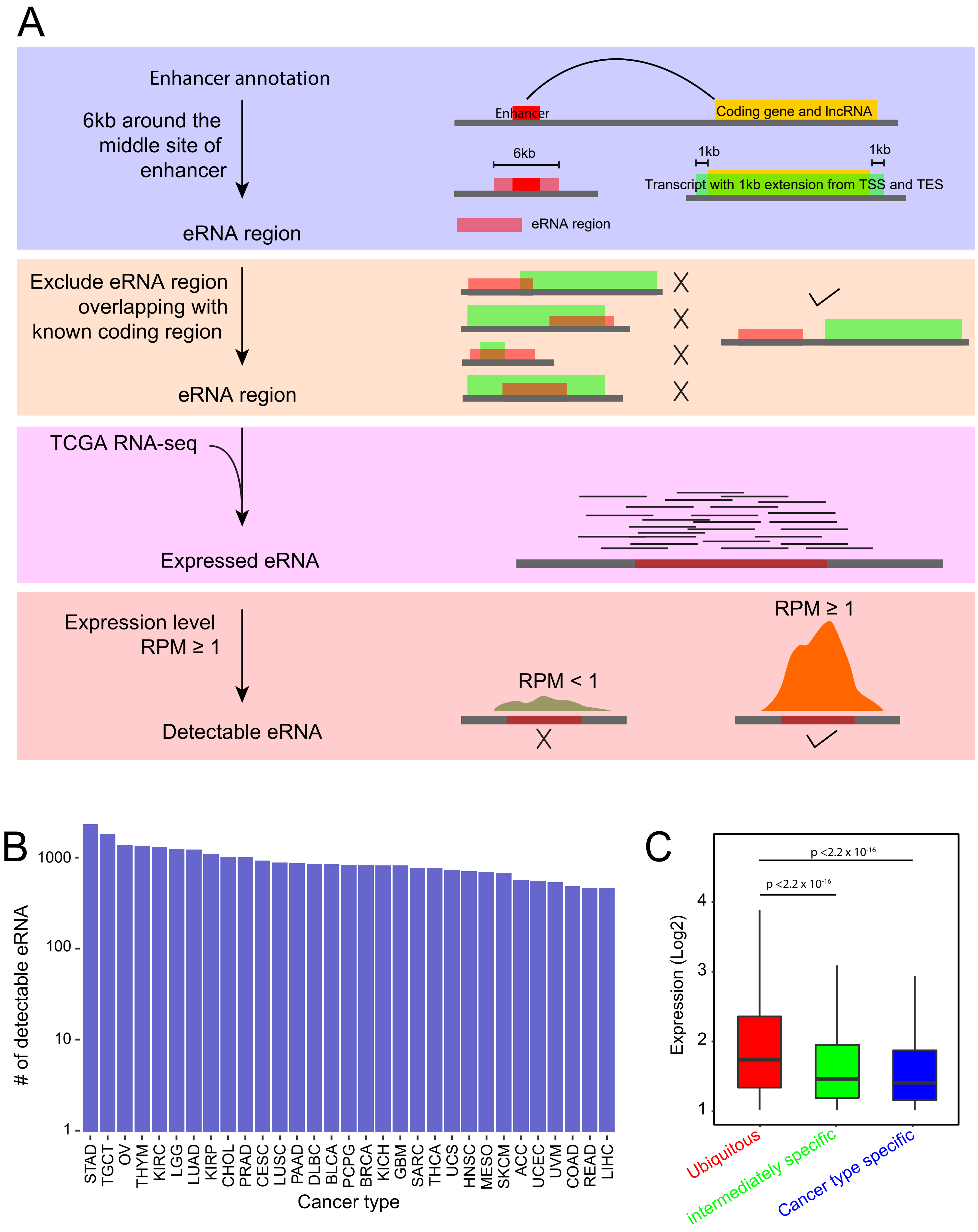

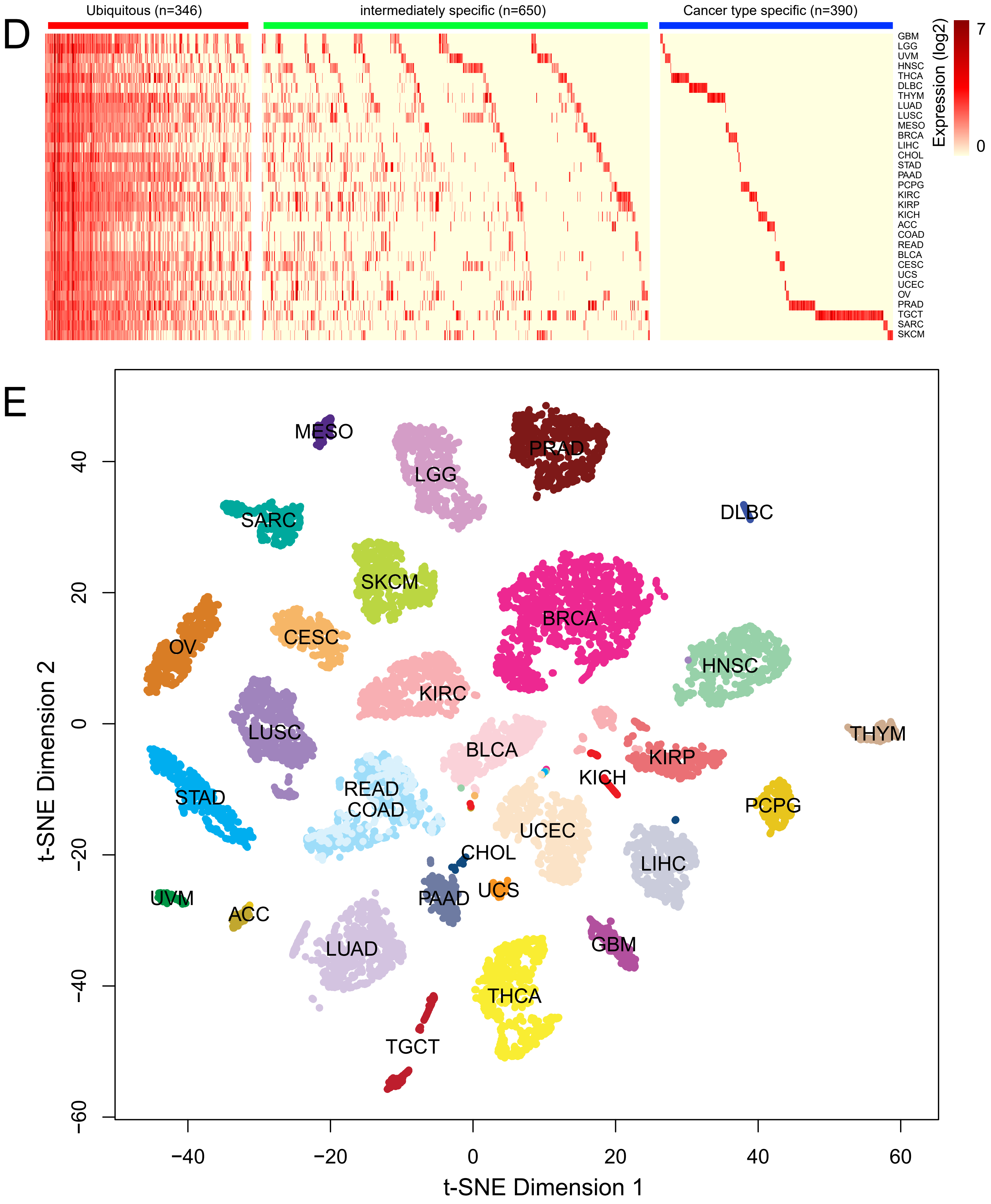

Supplementary Figure 1. Computational pipeline and features of detectable eRNAs across human cancers.
(A) Computational pipeline for detecting eRNAs. (B) Number of detectable eRNAs across human cancers. (C) Expression level of ubiquitous eRNAs, intermediately-specific eRNAs, and cancer-type–specific eRNAs. The boxes show the median ±1 quartile, with whiskers extending from the hinge to the smallest or largest value within 1.5 interquartile range from the box boundaries. (D) Expression profile of eRNA (average PRM ≥ 5) in human cancers. Scale bar denotes eRNA expression level. (E) The t-SNE analysis showed cancer-type specific pattern of eRNAs. Dots denote cancer samples and colors denote cancer types.

Supplementary Figure 2

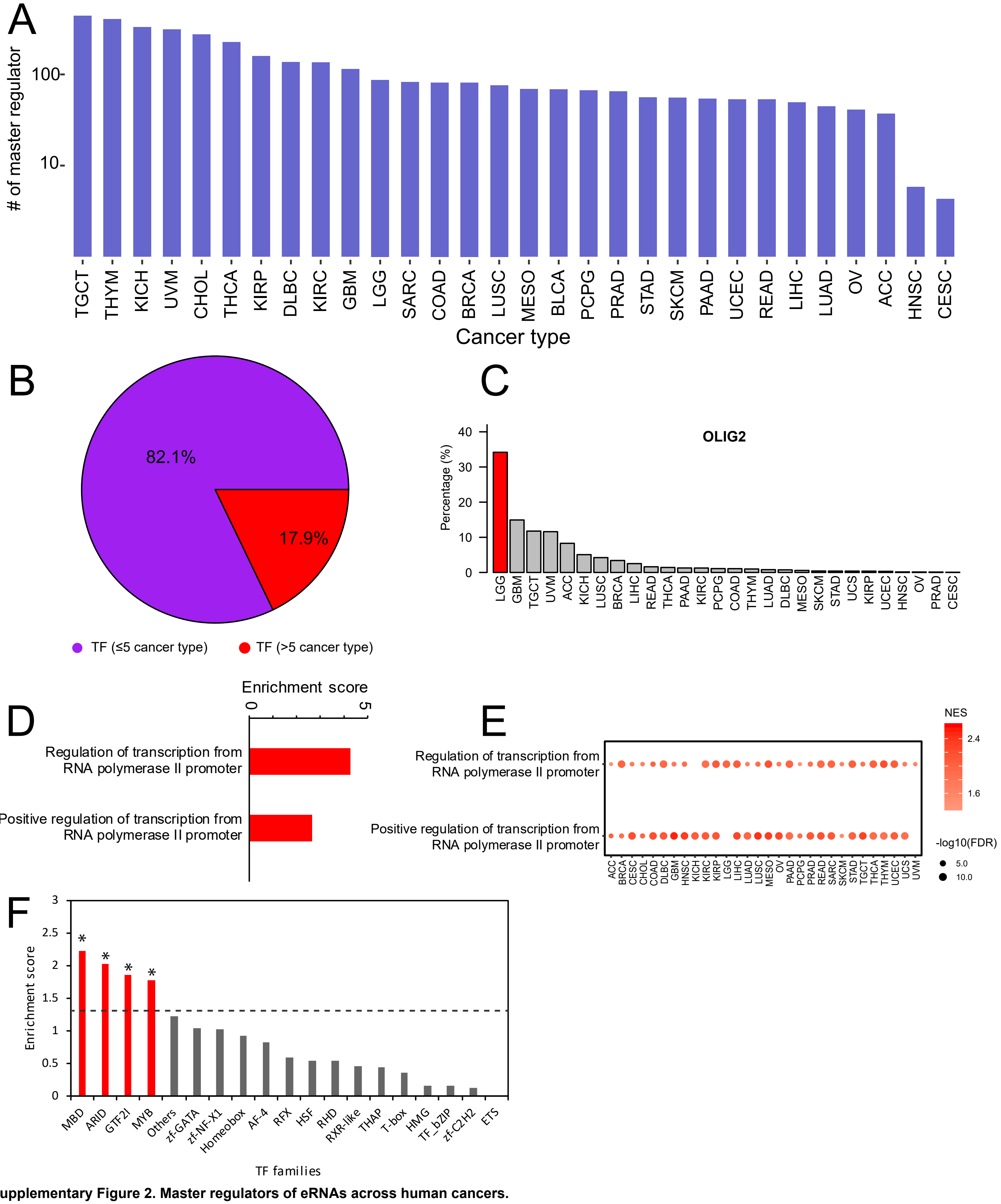

Supplementary Figure 2. Master regulators of eRNAs across human cancers.
(A) Number of putative master regulators of eRNAs across cancer types. (B) Overall cancer-type–specific feature for putative master regulators of eRNAs. (C) Percentage of eRNAs correlated to *OLIG2* across human cancers. (D) GO analysis for general putative master regulator analyzed by DAVID. (E) GO analysis for general master regulator analyzed by GSEA. Scale bar denotes normalized enrichment score (NES). (F) Enrichment analysis for general master regulators in 17 families.

Supplementary Figure 3

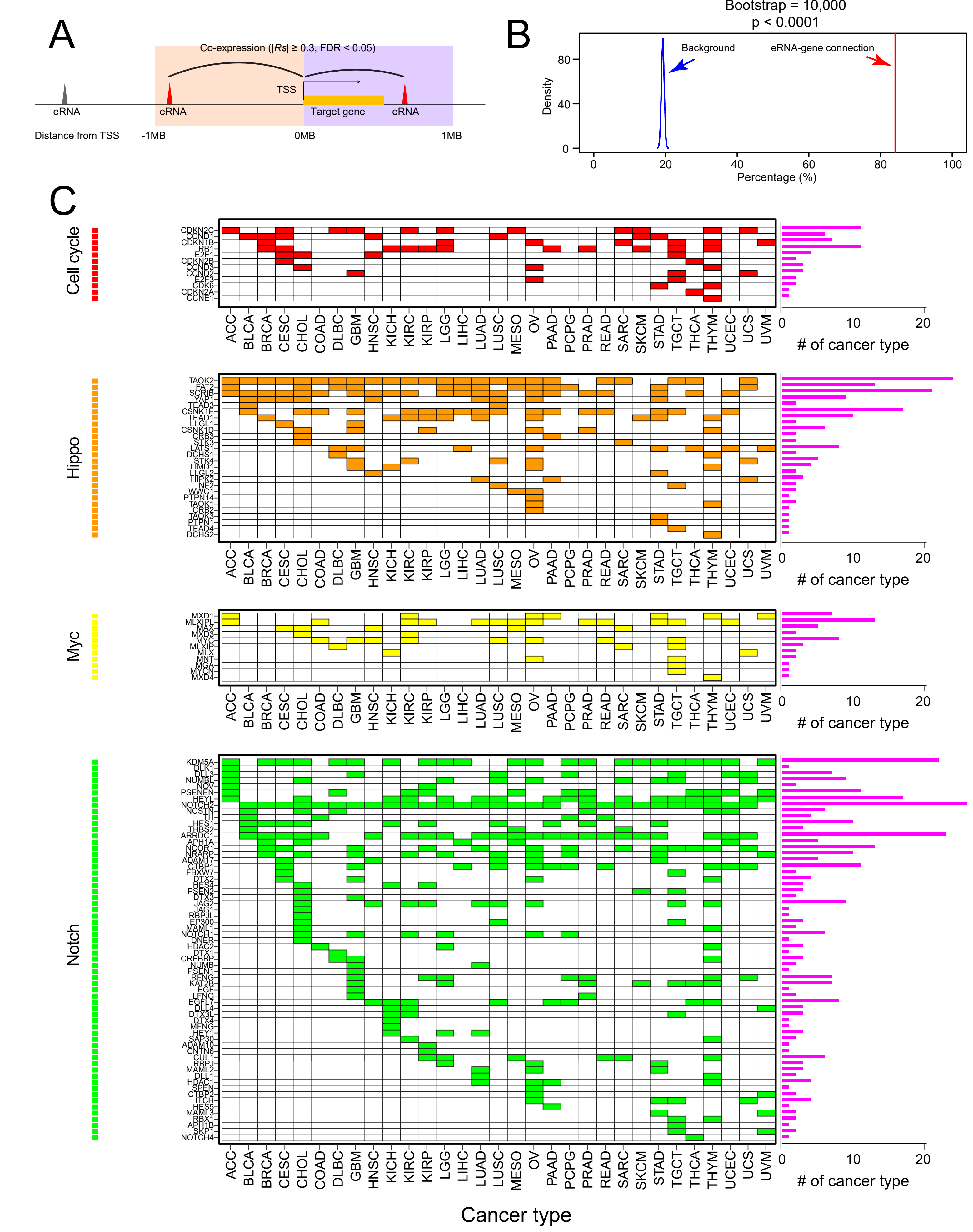

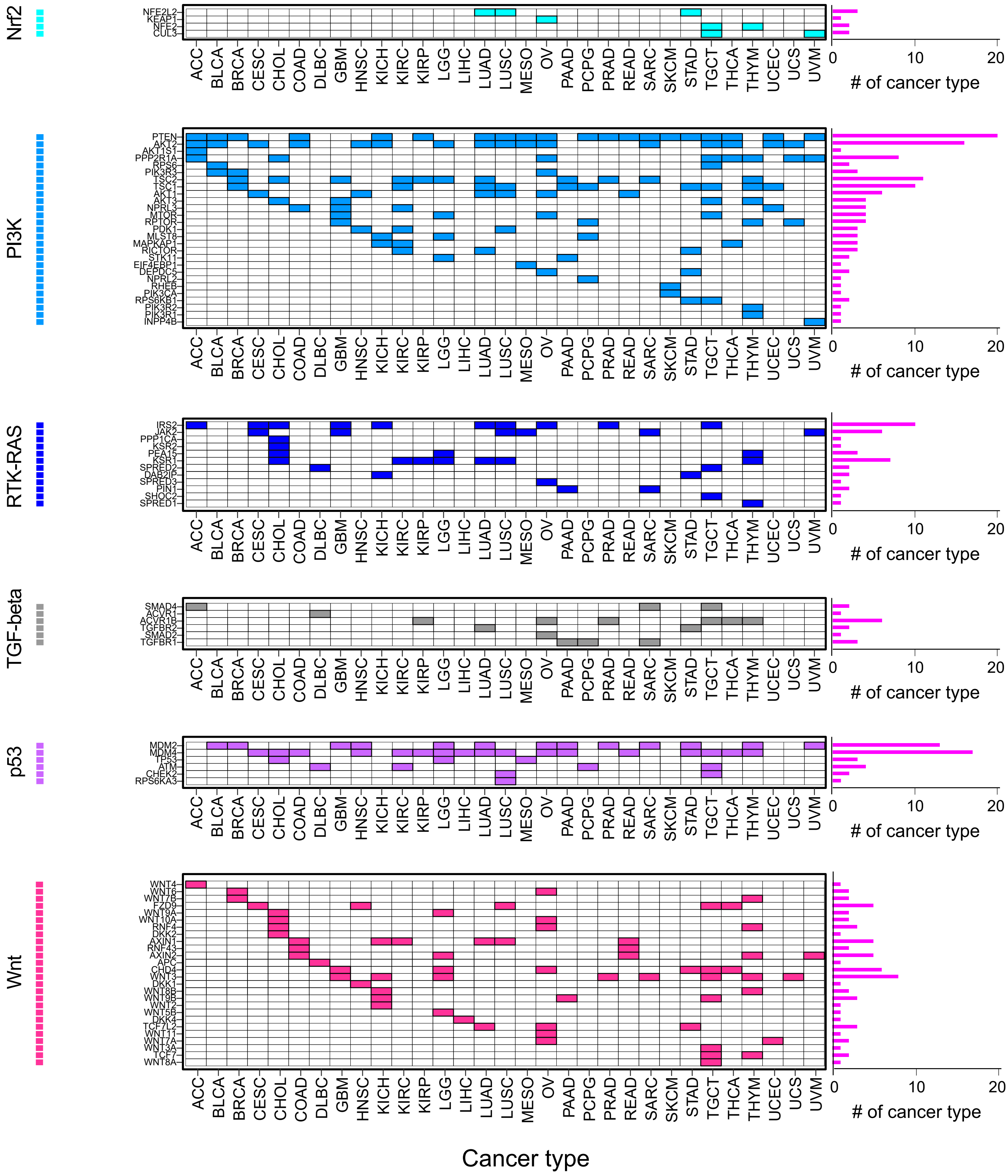

Nrf2
NFE2L2
KEAP1
NFE2
CUL3
PI3K
PTEN
AKT2
AKT1S1
PPP2R1A
RPS6
PIK3R3
TSC2
TSC1
AKT1
AKT3
NPRL3
MTOR
RPTOR
PDK1
MLST8
MAPKAP1
RICTOR
STK11
EIF4EBP1
DEPDC5
NPRL2
RHEB
PIK3CA
RPS6KB1
PIK3R2
PIK3R1
INPP4B
RTK-RAS
IRS2
JAK2
PPP1CA
KSR2
PEA15
KSR1
SPRED2
DAB2IP
SPRED3
PIN1
SHOC2
SPRED1
TGF-beta
SMAD4
ACVR1
ACVR1B
TGFBR2
SMAD2
TGFBR1
p53
MDM2
MDM4
TP53
ATM
CHEK2
RPS6KA3
Wnt
WNT4
WNT6
WNT7B
FZD9
WNT9A
WNT10A
RNF4
DKK2
AXIN1
RNF43
AXIN2
APC
CHD4
WNT3
DKK1
WNT8B
WNT9B
WNT2
WNT5B
DKK4
TCF7L2
WNT11
WNT7A
WNT3A
TCF7
WNT8A
ACC BLCA BRCA CESC CHOL COAD DLBC GBM HNSC KICH KIRC KIRP LGG LIHC LUAD LUSC MESO OV PAAD PCPG PRAD READ SARC SKCM STAD TGCT THCA THYM UCEC UCS UVM
0
10
20
# of cancer type
Cancer type

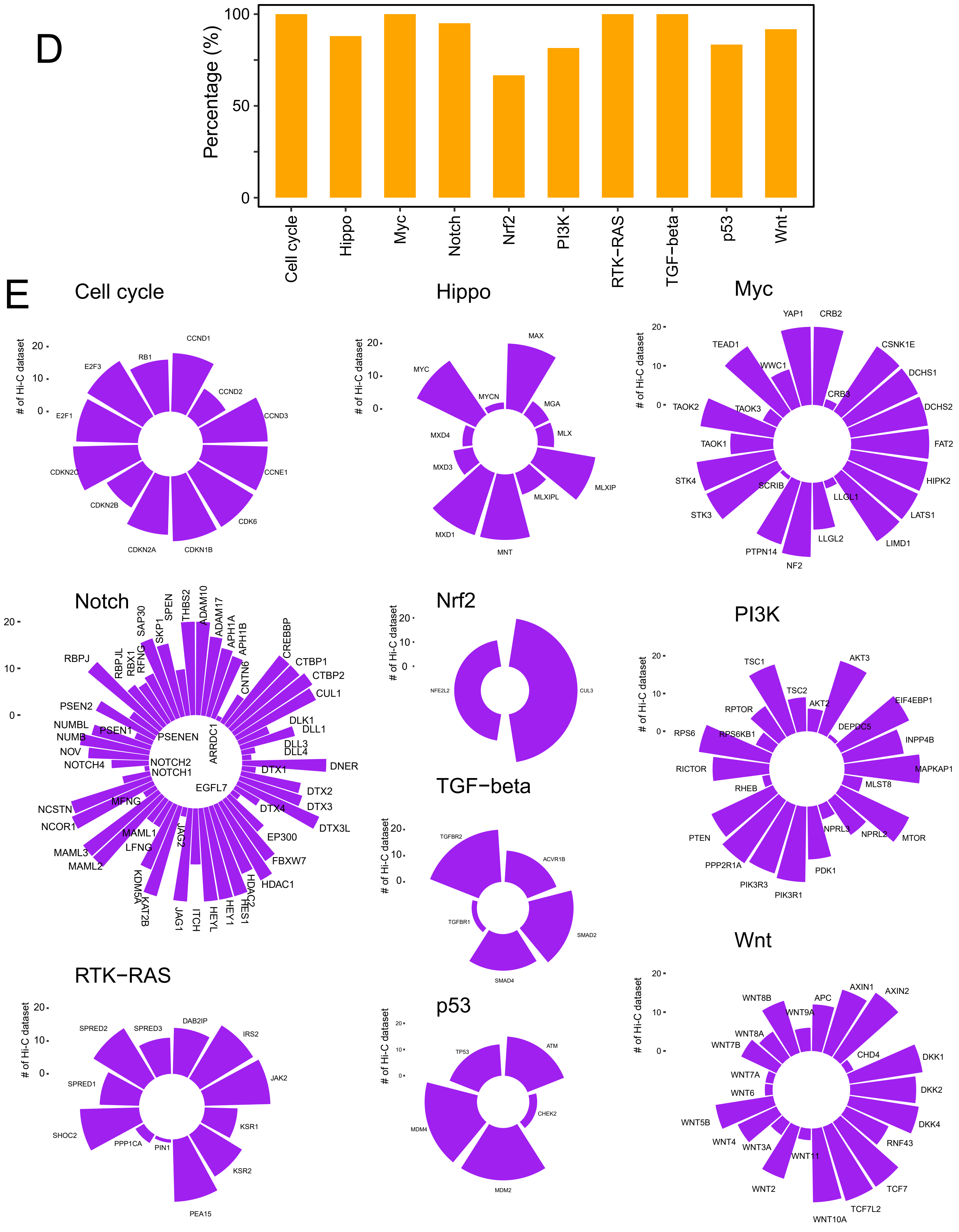
D
Percentage (%)
100
50
0
Cell cycle
Hippo
Myc
Notch
Nrf2
PI3K
RTK−RAS
TGF−beta
p53
Wnt
E
Cell cycle
# of Hi-C dataset
20
10
0
CCND1
RB1
E2F3
CCND2
CCND3
E2F1
CDKN2C
CCNE1
CDKN2B
CDK6
CDKN2A
CDKN1B
Hippo
YAP1
CRB2
TEAD1
WWC1
CSNK1E
DCHS1
CRB3
TAOK2
TAOK3
DCHS2
TAOK1
FAT2
STK4
SCRIB
HIPK2
LLGL1
STK3
LATS1
LLGL2
LIMD1
PTPN14
NF2
Myc
MAX
MYC
MYCN
MGA
MLX
MXD4
MXD3
MLXIP
MLXIPL
MXD1
MNT
Notch
RBPJ
RBPJL
RBX1
RFNG
SAP30
SKP1
SPEN
THBS2
ADAM10
ADAM17
APH1A
APH1B
CNTN6
CREBBP
CTBP1
CTBP2
CUL1
PSEN2
PSEN1
PSENEN
ARRDC1
DLK1
DLL1
DLL3
DLL4
NUMBL
NUMB
NOV
NOTCH4
NOTCH2
NOTCH1
DNER
DTX1
EGFL7
DTX2
DTX3
NCSTN
MFNG
DTX4
DTX3L
NCOR1
MAML1
JAG2
EP300
MAML3
LFNG
MAML2
FBXW7
HDAC1
KDM5A
KAT2B
JAG1
ITCH
HEYL
HEY1
HES1
HDAC2
Nrf2
NFE2L2
CUL3
TGF−beta
TGFBR2
ACVR1B
TGFBR1
SMAD2
SMAD4
PI3K
TSC1
AKT3
TSC2
AKT2
EIF4EBP1
RPTOR
DEPDC5
RPS6
RPS6KB1
INPP4B
RICTOR
MAPKAP1
RHEB
MLST8
PTEN
NPRL3
NPRL2
MTOR
PPP2R1A
PDK1
PIK3R3
PIK3R1
RTK−RAS
DAB2IP
SPRED2
SPRED3
IRS2
JAK2
SPRED1
KSR1
SHOC2
PPP1CA
PIN1
KSR2
PEA15
p53
TP53
ATM
CHEK2
MDM4
MDM2
Wnt
AXIN1
AXIN2
WNT8B
APC
WNT9A
WNT8A
WNT7B
CHD4
DKK1
WNT7A
WNT6
DKK2
WNT5B
DKK4
WNT4
WNT3A
RNF43
WNT11
WNT2
TCF7
TCF7L2
WNT10A

F

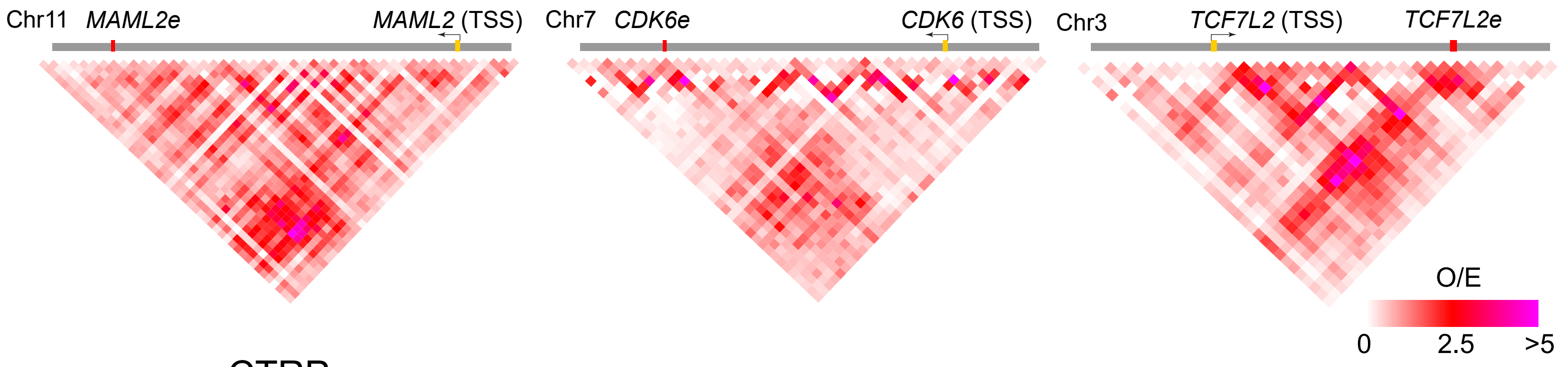
Chr11 MAML2e
MAML2 (TSS)
Chr7 CDK6e
CDK6 (TSS)
Chr3
TCF7L2 (TSS)
TCF7L2e
O/E
0
2.5
>5

G

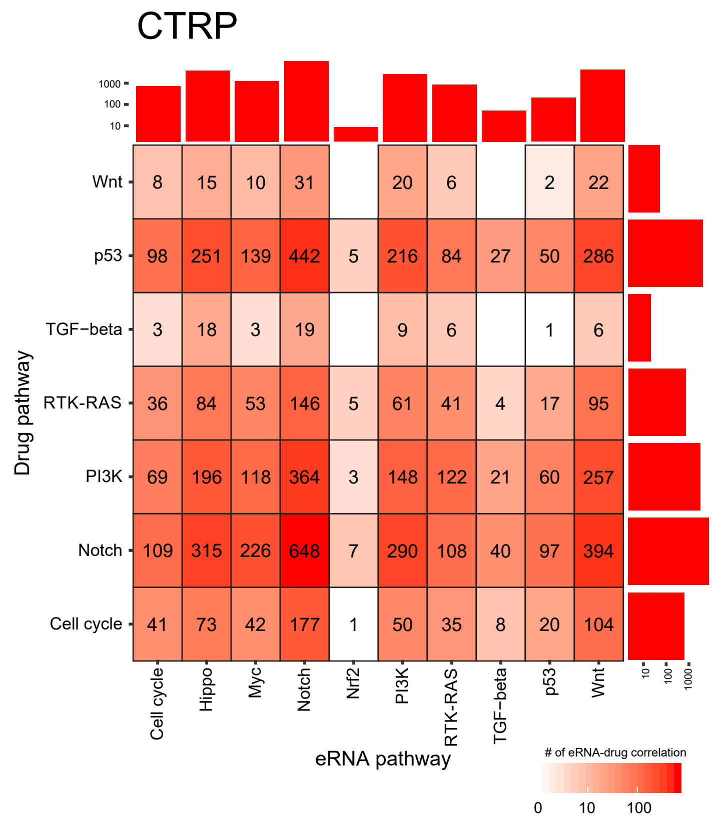
CTRP
1000
100
10
Drug pathway
Wnt
p53
TGF−beta
RTK-RAS
PI3K
Notch
Cell cycle
8 15 10 31 20 6 2 22
98 251 139 442 5 216 84 27 50 286
3 18 3 19 9 6 1 6
36 84 53 146 5 61 41 4 17 95
69 196 118 364 3 148 122 21 60 257
109 315 226 648 7 290 108 40 97 394
41 73 42 177 1 50 35 8 20 104
Cell cycle
Hippo
Myc
Notch
Nrf2
PI3K
RTK-RAS
TGF−beta
p53
Wnt
10
100
1000
eRNA pathway
# of eRNA-drug correlation
0
10
100

H

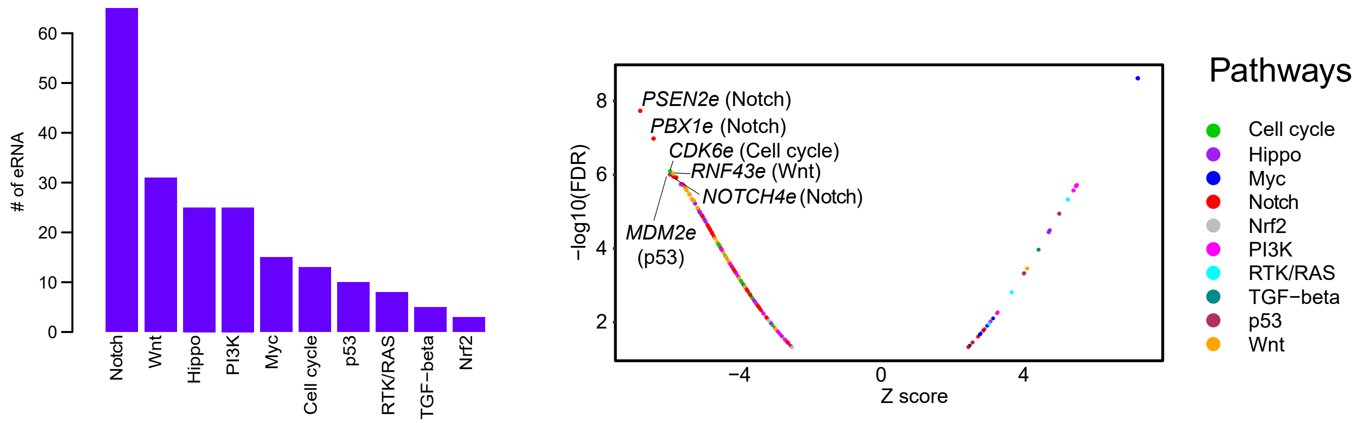
# of eRNA
60
50
40
30
20
10
0
Notch
Wnt
Hippo
PI3K
Myc
Cell cycle
p53
RTK/RAS
TGF−beta
Nrf2
−log10(FDR)
8
6
4
2
PSEN2e (Notch)
PBX1e (Notch)
CDK6e (Cell cycle)
RNF43e (Wnt)
NOTCH4e (Notch)
MDM2e
(p53)
−4
0
4
Z score
Pathways
Cell cycle
Hippo
Myc
Notch
Nrf2
PI3K
RTK/RAS
TGF−beta
p53
Wnt

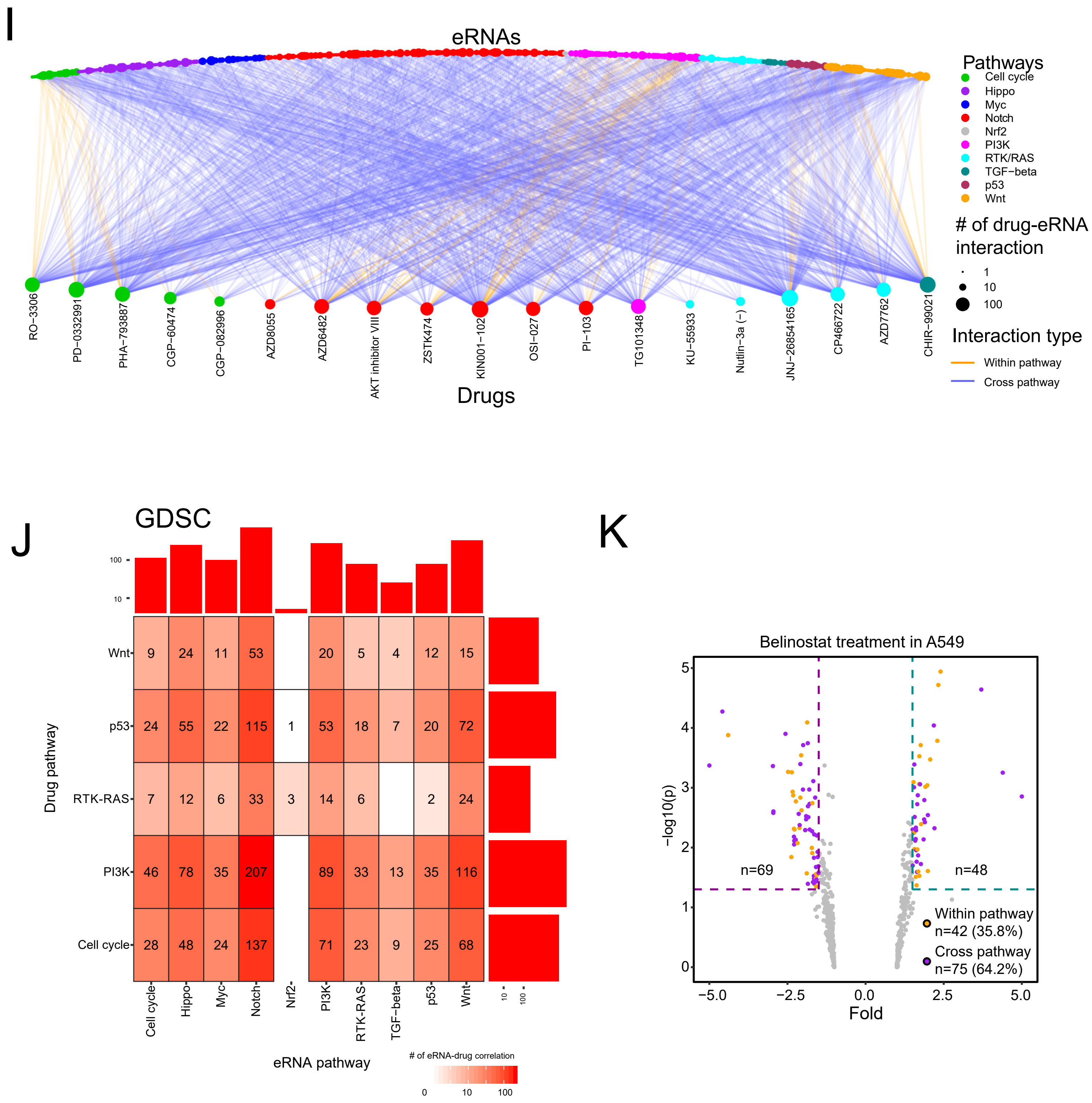

Supplementary Figure 3. eRNA putative target genes and drug-associated eRNAs.

(A) Definition for eRNA putative target genes. (B) Permutation test of Hi-C interactions between eRNA-gene correlated pairs and genome background of random eRNA-gene pairs across 20 human tissues. (C) Landscape of eRNA putative target genes in 10 cancer signaling pathways across human cancers. (D) Percentage of Hi-C interaction between eRNAs and putative target genes in 10 cancer signaling pathways. X-scale denotes pathways and y-scale denotes percentage of eRNA-gene association has Hi-C interaction. (E) Hi-C interaction between eRNAs and putative target genes for each cancer signaling pathway. Pie charts represent the number of dataset with interaction (O/E > 1) in Hi-C data, while the y-scale (0, 10 and 20) means number of datasets showed interaction (O/E > 1) in Hi-C data. (F) Example for Hi-C interaction between cancer signaling genes *MAML2* (Notch), *CDK6* (Cell cycle), *TCF7L2* (Wnt) and their eRNAs in adrenal, lung, and liver, respectively. Scale bar denotes Hi-C O/E value. (G) Statistical analyses for drug-eRNA association across cancer signaling pathways in CTRP database. Scale bar denotes number of eRNA-drug correlation. (H) Distribution of belinostat correlated eRNAs in cancer signaling pathways. (I) Associations between drugs and eRNAs across cancer signaling pathways in GDSC. (J) Statistical analyses for drug-eRNA association across cancer signaling pathways in GDSC database. Scale bar denotes number of eRNA-drug correlation. (K) Alterations of eRNAs under belinostat treatment in A549 cells.

Supplementary Figure 4

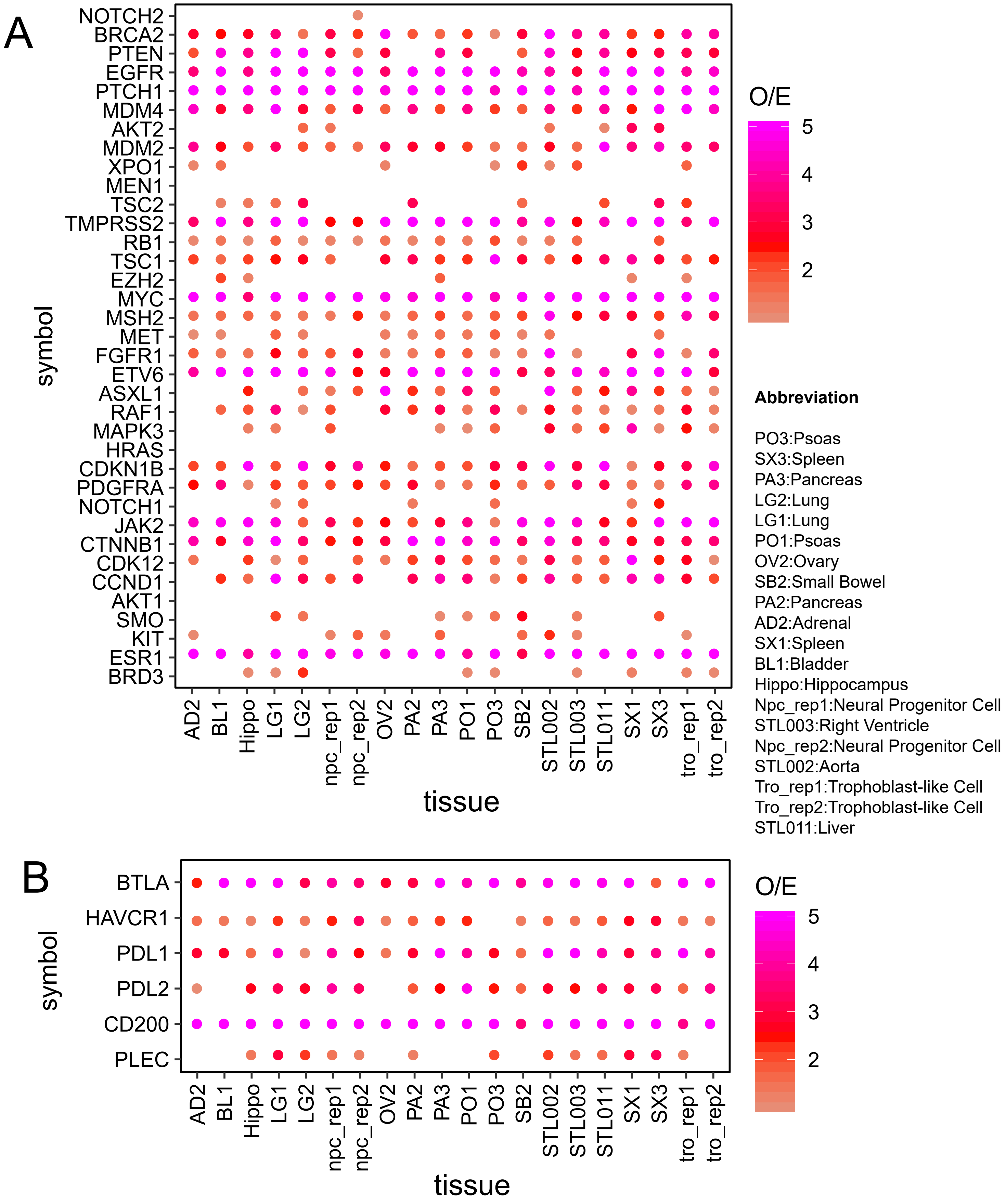

Supplementary Figure 4. Hi-C interaction between eRNAs and clinically actionable genes and immune checkpoints across tissues. (A) Hi-C interaction between clinically actionable genes and eRNAs. (B) Hi-C interaction between immune checkpoints and eRNAs. Scale bars denote Hi-C O/E value.

Supplementary Figure 5

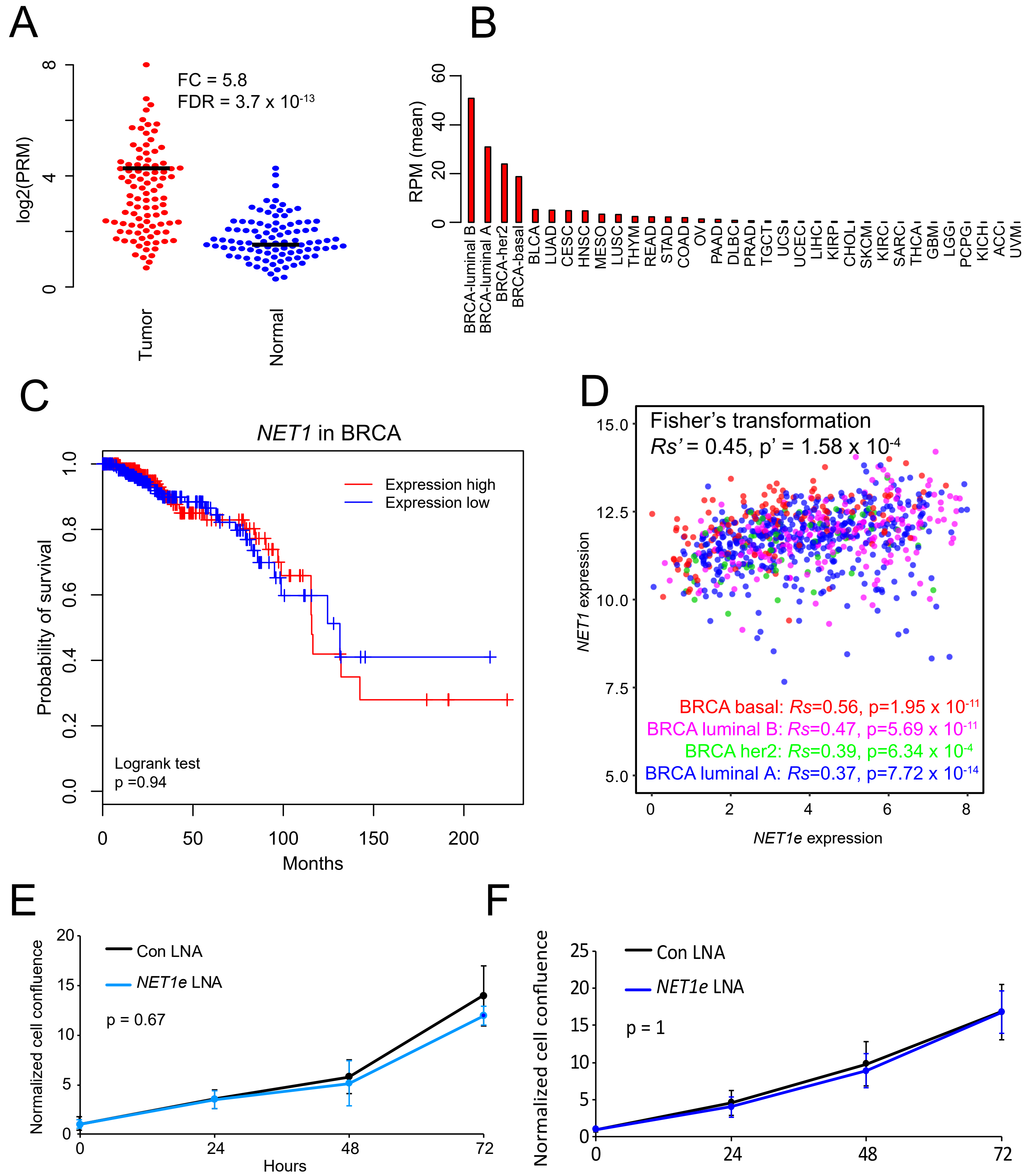

Supplementary Figure 5. Features and functional characterization of *NET1e*.
(A) Expression alteration of *NET1e* in paired BRCA samples (n = 99). (B) Expression of *NET1e* in human cancers, sample sizes for BRCA subtypes are Luminal B = 215, Luminal A = 486, HER2 = 77, and basal = 161. (C) Survival curves for *NET1* in BRCA. (D) Spearman's correlation between *NET1e* and *NET1* in each BRCA subtype. (E) Cell growth assay by knockdown *NET1e* in MCF10A cells. (F) Cell growth assay by knockdown *NET1e* in Hela cells. Error bars in E and F indicate mean ± SD.

Supplementary Figure 6

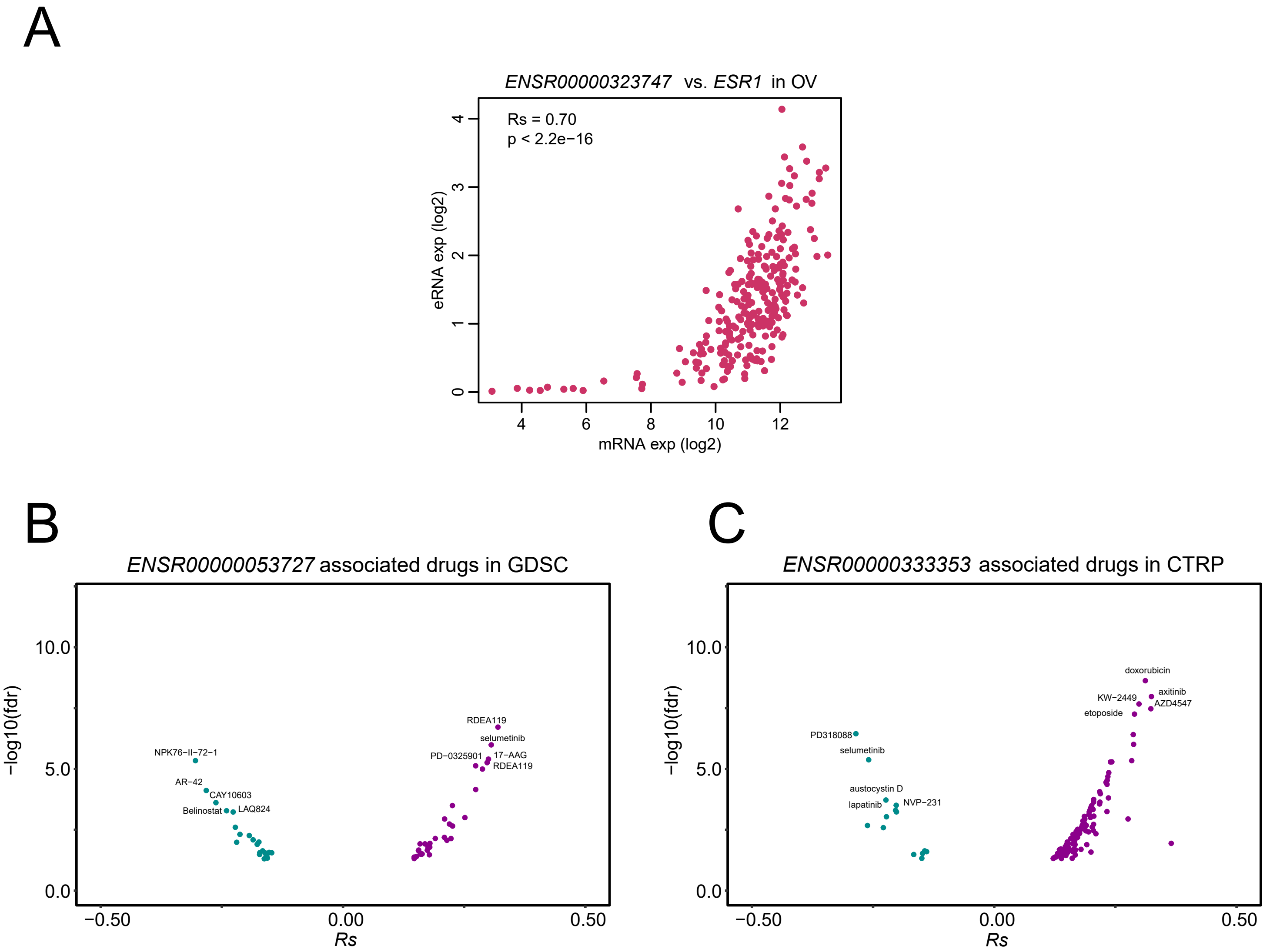

Supplementary Figure 6. Overview of eRic.
(A) Associations between eRNAs and putative target genes. (B-C) Associations between eRNAs and drugs in CTRP (B) and GDSC (C).

Supplementary Figure 7

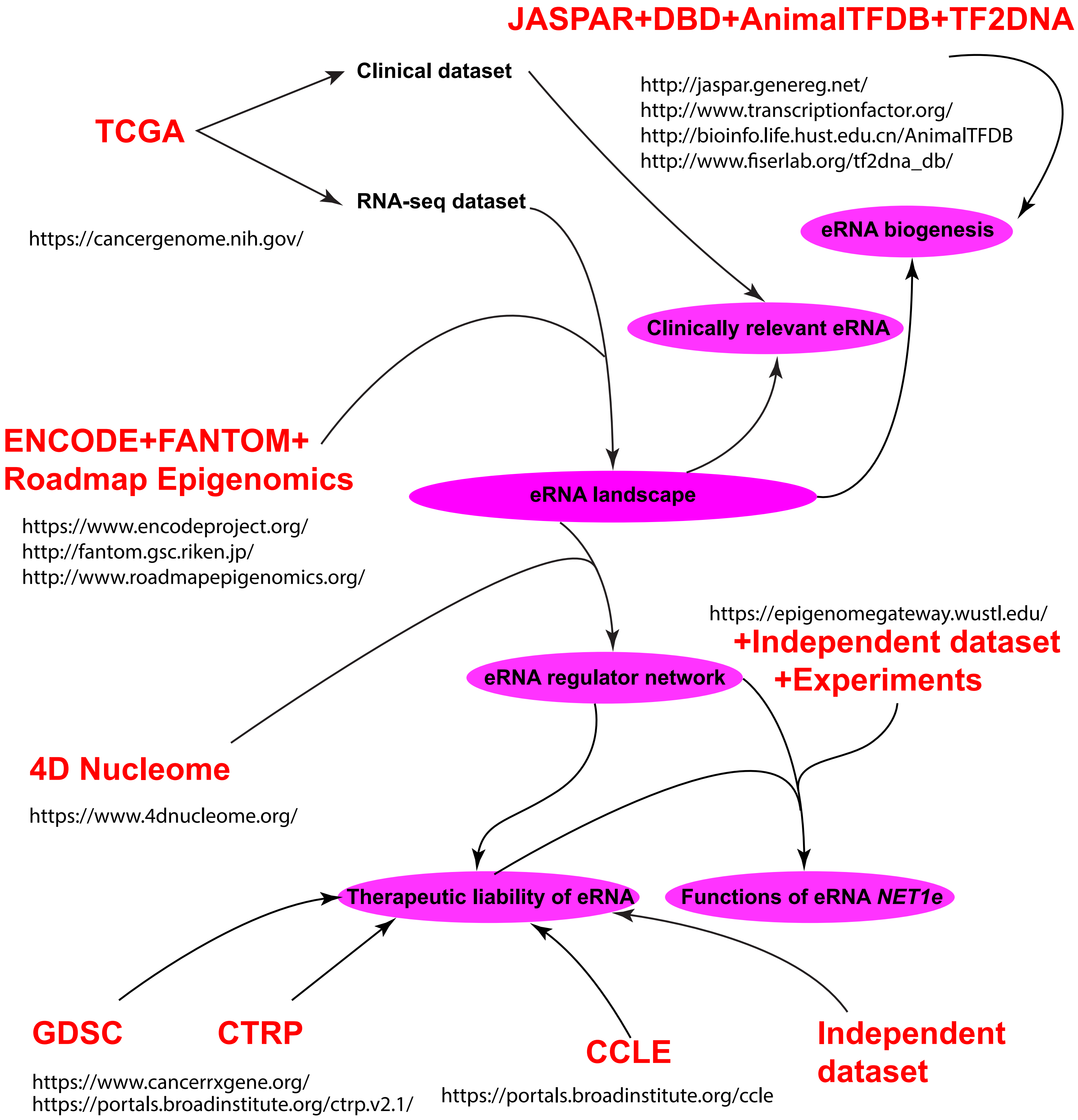

Supplementary Figure 7. Multi-omics data and pharmacogenomics data used in this study.

Supplementary Tables

Supplementary Table 1. List of multi-omics data and pharmacogenomics data used in this study.

Dataset	Description	Link
TCGA RNA-seq	Genomic Data	https://portal.gdc.cancer.gov/
TCGA clinical dataset	Clinical data	https://portal.gdc.cancer.gov/
Ensembl Regulator Build	Genomic annotation	https://useast.ensembl.org/index.html
Fantom enhancer	Genomic annotation	http://fantom.gsc.riken.jp/index.html
Roadmap Epigenomics	Genomic annotation	http://www.roadmapepigenomics.org/
Jaspar	TF gene list	http://jaspar.genereg.net/
TF2dna	TF gene list	http://www.fiserlab.org/tf2dna_db/
AnimalTFDB	TF gene list	http://bioinfo.life.hust.edu.cn/AnimalTFDB/
DBD	TF gene list	http://www.transcriptionfactor.org/index.cgi/Home
CCLE RNA-seq	Genomic Data	https://portals.broadinstitute.org/ccle
CTRP drug response	Drug response data	https://portals.broadinstitute.org/ctrp/
GDSC drug response	Drug response data	https://www.cancerrxgene.org/
Human Hi-C interaction	Genomic Data	http://promoter.bx.psu.edu/public/HiCPlus/matrix/
MCF7 Gro-seq	Genomic Data	https://epigenomegateway.wustl.edu/
MCF7 ChIP-seq	Genomic Data	https://epigenomegateway.wustl.edu/

Supplementary Table 2. CRISPR gRNA, LNA, and PCR primers used in this study.

Name	Sequences
Control LNA	TTACAGAAGGAGTCGT
NET1e LNA#1	TTGAAGCTGGTGGAGC
NET1e LNA#2	GAAGAACCTCTTGGAA
NET1e LNA#3	Qiagen LG00000002
NET1e gRNA1-Fwd	CACCGGAGGAATGTGCAAACGGCCC
NET1e gRNA1-Rev	AAACGGGCCGTTTGCACATTCCTCC
NET1e gRNA2-Fwd	CACCGAGCCTACACCCTGGAGGTAC
NET1e gRNA2-Rev	AAACGTACCTCCAGGGTGTAGGCTC
NET1e-Fwd	CAGGCCTACCAGGATGGATA
NET1e-Rev	AGTTGACTTGGGTGGGACAG
NET1-Fwd	ACCTGGATGAAAAGCAGAGG
NET1-Rev	GGTAAGAGTGCCGTTCGTTC
GAPDH-Fwd	GTTTTTCTAGACGGCAGGTCA
GAPDH-Rev	AACATCATCCCTGCCTCTACT
