# Supplementary material for: Transcriptional landscape and clinical utility of enhancer RNAs for eRNA-targeted therapy in cancer
Source: Nat Commun. 2019 Oct 8;10:4562. doi: 10.1038/s41467-019-12543-5 (PMC6783481; doi:10.1038/s41467-019-12543-5)
Supplement: Supplementary file 2 — Description of Additional Supplementary Files [file 41467_2019_12543_MOESM2_ESM.pdf]

Description of Additional Supplementary Data

Supplementary Data 1. Number of samples, detectable eRNAs, and detail for abbreviation of each cancer type.

Supplementary Data 2. Putative master regulators for eRNAs in each cancer type.

Supplementary Data 3. Spearman's correlation between eRNAs and target genes in cancers.

Supplementary Data 4. Spearman's correlation and Hi-C interactions between eRNAs and clinically actionable genes.

Supplementary Data 5. Spearman's correlation and Hi-C interactions between eRNAs and immune checkpoints.

Supplementary Data 6. Expression alterations of eRNAs across cancer types.

Supplementary Data 7. List of drugs correlated to *NET1e* from CTRP database and GDSC database.

Supplementary Data 8. Clinically relevant eRNAs across cancer types.
